# Profiling the B/T cell receptor repertoire of lymphocyte derived cell lines

**DOI:** 10.1186/s12885-018-4840-5

**Published:** 2018-10-01

**Authors:** Kar-Tong Tan, Ling-Wen Ding, Qiao-Yang Sun, Zhen-Tang Lao, Wenwen Chien, Xi Ren, Jin-Fen Xiao, Xin Yi Loh, Liang Xu, Michael Lill, Anand Mayakonda, De-Chen Lin, Henry Yang, H. Phillip Koeffler

**Affiliations:** 1 Cancer Science Institute of Singapore, National University of Singapore, Singapore, Singapore; 2 Department of Haematology, Singapore General Hospital, Singapore, Singapore; 3 Division of Hematology/Oncology, Cedars-Sinai Medical Center, UCLA School of Medicine, Los Angeles, USA

**Keywords:** BCR/TCR receptor repertoire, EBV lymphocytes, Cancer cell lines

## Abstract

**Background:**

Clonal VDJ rearrangement of B/T cell receptors (B/TCRs) occurring during B/T lymphocyte development has been used as a marker to track the clonality of B/T cell populations.

**Methods:**

We systematically profiled the B/T cell receptor repertoire of 936 cancer cell lines across a variety of cancer types as well as 462 Epstein-Barr Virus (EBV) transformed normal B lymphocyte lines using RNA sequencing data.

**Results:**

Rearranged B/TCRs were readily detected in cell lines derived from lymphocytes, and subclonality or potential biclonality were found in a number of blood cancer cell lines. Clonal BCR/TCR rearrangements were detected in several blast phase CML lines and unexpectedly, one gastric cancer cell line (KE-97), reflecting a lymphoid origin of these cells. Notably, clonality was highly prevalent in EBV transformed B lymphocytes, suggesting either transformation only occurred in a few B cells or those with a growth advantage dominated the transformed population through clonal evolution.

**Conclusions:**

Our analysis reveals the complexity and heterogeneity of the BCR/TCR rearrangement repertoire and provides a unique insight into the clonality of lymphocyte derived cell lines.

**Electronic supplementary material:**

The online version of this article (10.1186/s12885-018-4840-5) contains supplementary material, which is available to authorized users.

## Background

Clonal V(D)J [variable (V), diversity (D) and joining (J)] rearrangement which occurs during development of B/T lymphocytes has been used as a marker to track the clonality of B/T cell populations [1, 2]. This approach is feasible because lymphoid neoplasm/lymphoproliferative cells originate and expand from a single cell; and the progeny cells share the same VDJ rearrangement. A pattern of a monoclonal/oligoclonal population (manifested as the over-representation of either one or a few uniquely rearranged sequences) suggests the presence of a lymphoid neoplasm [or non-malignant clonal lymphoproliferative disorder, such as monoclonal B cell lymphocytosis [3] or monoclonal gammopathy of undetermined significance (MGUS)].

In this study, we systematically profiled the B/T cell receptor repertoire of 936 cancer cell lines across a variety of cancer types, as well as 462 Epstein-Barr Virus (EBV) transformed normal B lymphocyte lines, using RNA sequencing data from the Cancer Cell Line Encyclopedia (CCLE) [4] and Geuvadis RNA sequencing project of 1000 Genomes samples [5]. This study cohort contains cell lines from a variety of solid tumors and 164 blood cancer cell lines (annotated as haematopoietic and lymphoid tissue in CCLE), as well as immortalized “normal” B-lymphocyte cell lines. Cancer cell lines are typically deemed to be “pure”, due to the lack of normal stroma cells and infiltrating T/B cells which are frequently presented in primary tumor samples; thus, this cell line collection provides a unique opportunity to profile faithfully and comprehensively the immunoglobulin/TCR gene rearrangement events in different types of blood cancers.

## Methods

Transcriptome sequencing data were downloaded from the CCLE and Geuvadis RNA sequencing databases; and the B/T cell receptor repertoire of each cell line was analyzed using MiXCR [6]. The 936 CCLE cancer cell lines were authenticated before uniformly processed RNA sequencing (paired-end 100 × 2 bp) [4]. The growth and uniformly processed RNA sequencing (paired-end 75 × 2 bp) of 462 Epstein-Barr Virus (EBV) transformed normal B lymphocyte lines were described in ref [5]. The B cell and T cell gene expression signature and expression of lineage specific markers (e.g., CD4/CD8 for T cell and CD19/CD20 for B cells) were analyzed from the cell line microarray expression data. The relative clonal CPM value (count per million RNA sequencing reads) was calculated by dividing the clonal read counts by the total RNA sequencing read counts. Each clonotypes were determined based on a unique nucleotide sequence of the VDJ junction (which codes for the CDR3 region) and supported by ≥30 sequencing reads. Two clonotypes have the same rearrangement pattern but have ≥1 bp difference in their nucleotide sequence VDJ junction were considered as different clonotypes. As we cannot exclude the possibility of expression of biallelic rearrangements (where the second allele is usually non-productive or has markedly decreased expression), we refer to bi/oligoclonality as the observation of more than two/four clonotypes within the same type of rearrangement within a cell line [7] (either ≥3 IGH or ≥ 3 IGL or ≥ 3 TRAV, or > 4 IGKV or > 4 TRBV [7]). For example, a cell line with three different IGH rearrangements (e.g., 60% of IGHV6–1-IGHD1–20-IGHJ4, 20% IGHV3–23-IGHD2–8-IGHJ6 and 18% of IGHV3–20-IGHD2–8-IGHJ5) will be regarded as potential bi/oligoclonality. Nonproductive rearrangement was referred as out of frame rearrangement (denoted with “_” in the CDR sequence) or rearrangement with stop codon inside the CDR3 region (denoted with “*” in the CDR3 sequence).

## Results

### BCR repertoire of cancer cell lines derived from B lymphocytes

**Fig. 1:**
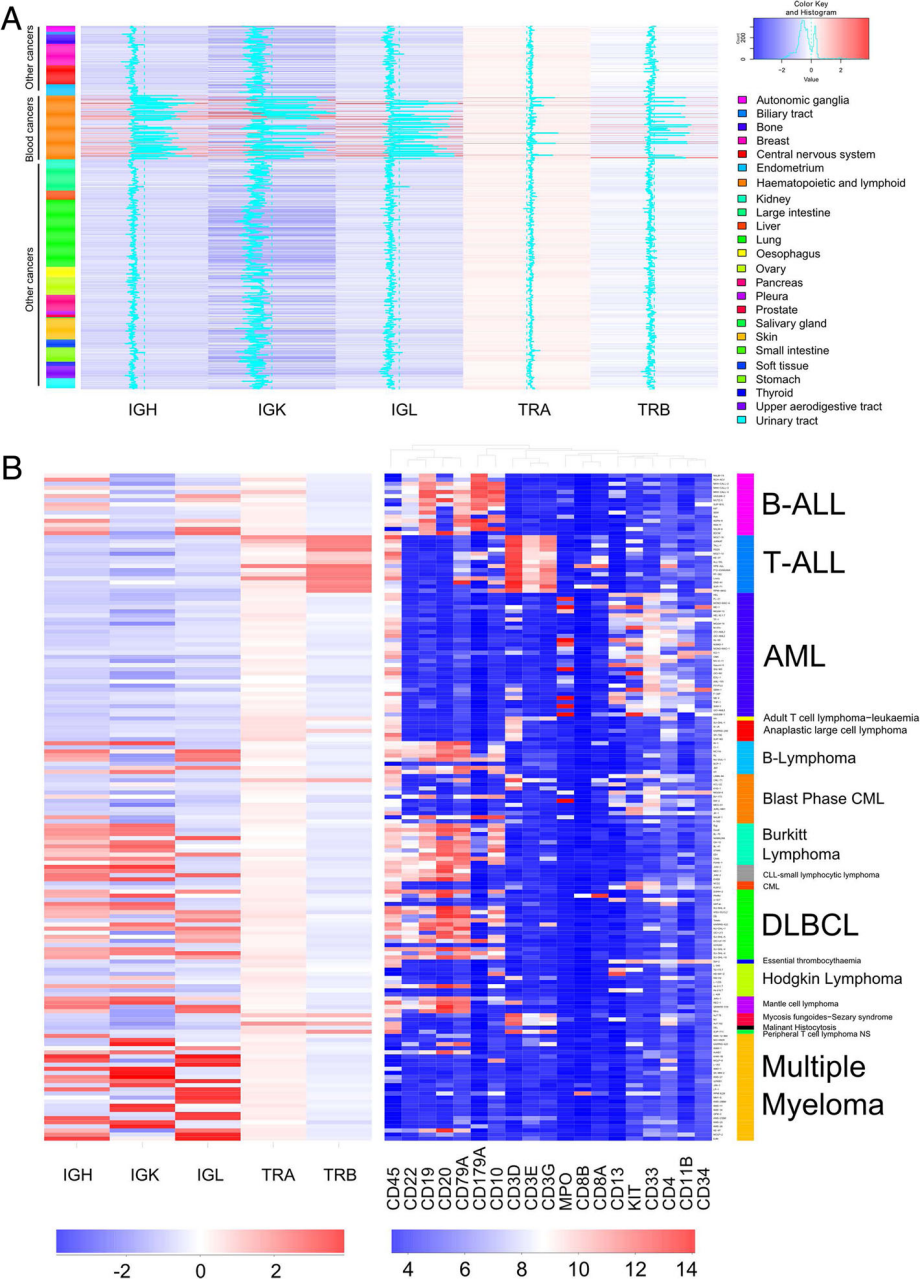
Heatmap of the BCR/TCR rearrangement pattern of cancer cell lines. **a**. Heatmap of the clonal rearrangement pattern of IGH, IGK/L and TRA/B in 936 cancer cell lines. The histogram indicates the number of samples with the given expression level. The line in the heatmap represents the gene expression of the particular sample, with a line drawn more towards the right, indicating a higher level of expression. **b**. Heatmap of the clonal rearrangement pattern of IGH, IGK/L and TRA/B, as well as the expression of the cell surface hematopoietic lineage marker in 164 blood cancer cell lines. Values were normalized with either z score (left panel) or shown as log2 expression (right panel)

**Fig. 2:**
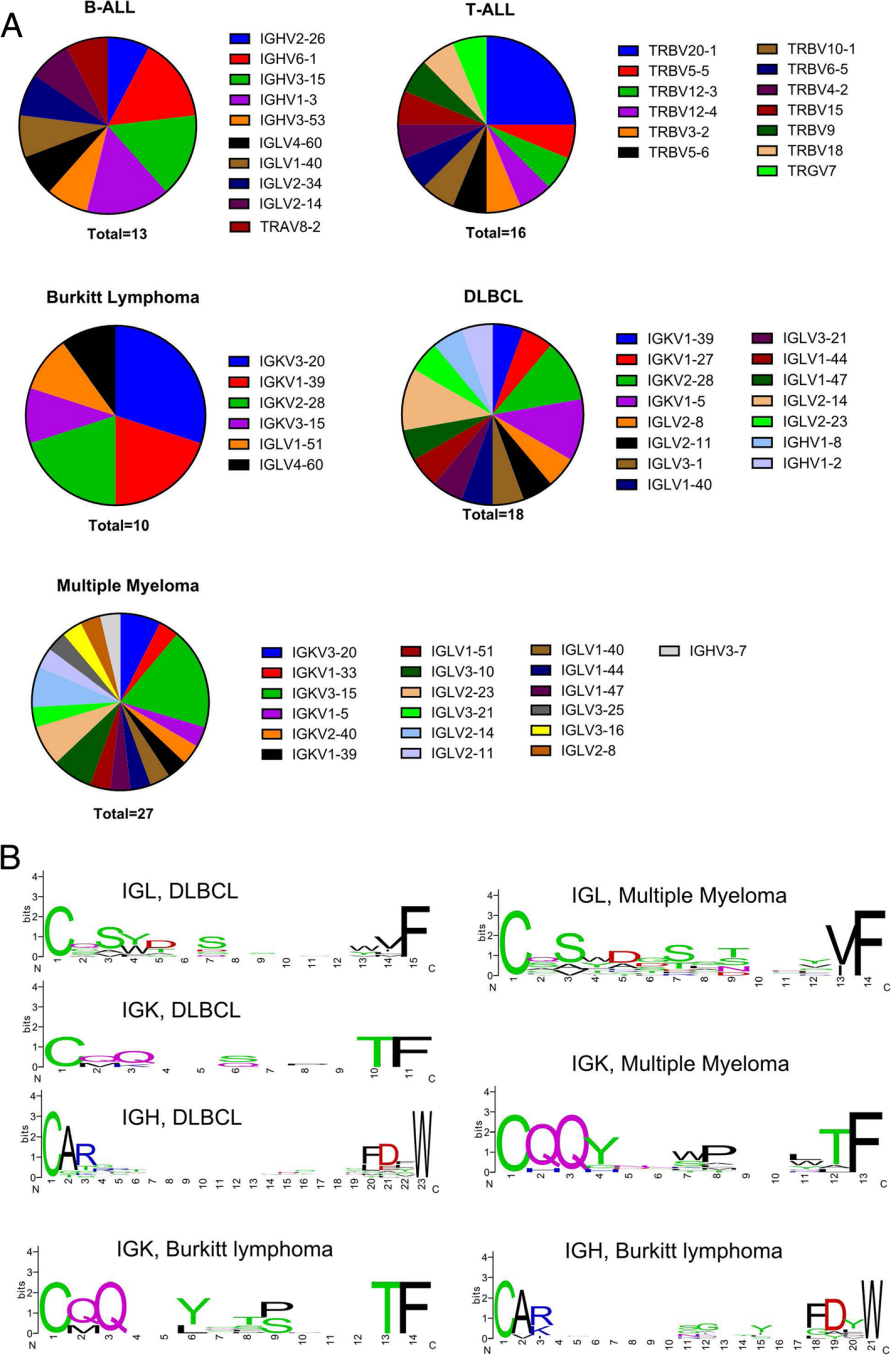
IGHV, IGK/L V segment usage and consensus sequence of CDR3 region of blood cancer cell lines. **a**. IGHV and IGK/L V segment usage of the highest expressed rearrangement of B-ALL, T-ALL, Burkitt lymphoma, diffuse large B cell lymphoma (DLBCL) and multiple myeloma. **b**. Consensus sequence of CDR3 region of the dominant rearrangement in multiple myeloma, DLBCL and Burkitt lymphoma (left panel). CDR3 region sequences were aligned using ClustalW and the consensus sequences were plotted using weblogo (right panel)

Cancer cell lines derived/transformed from mature B cells that have undergone BCR selection include multiple myeloma (*n* = 25), mantle cell lymphoma (*n* = 4), Burkitt lymphoma (*n* = 10) and chronic lymphocytic leukemia-small lymphocytic lymphoma (CLL, n = 4) etc. High expression of both IGH (heavy chain) and IGK/L (light chain) rearrangement are detected in all of the mantle cell lymphoma, Burkitt lymphoma and CLL-small lymphocytic lymphoma cell lines (Fig. 1). In multiple myeloma (MM), except for cell line KMS-12-BM [in which only a productive IGH rearrangement (IGHV3–7-IGHD4–23-IGHJ6, 18,169 reads) is dominantly expressed], many of the MM cell lines (Additional file 1: Tables S1 and S2) express a single dominant IGK or IGL rearrangement accompanying an either extremely low (< 30 RNA sequencing reads) or a completely undetectable heavy chain (IGH) rearrangement (Fig. 2), suggesting that these cell lines (NCI-H929, KARPAS-620, L363, SK-MM-2, JJN3, RPMI8226, KMS-28BM, KMS-11, KMS34, OPM-2, KMS-26) likely belong to light chain multiple myeloma (LC-MM) [8], a poor prognosis MM subtype constituting 15% of myelomas which are characterized by either deletion or silencing of the IGH loci after VDJ rearrangement [8]. While the majority of multiple myeloma cell lines express either a single dominant IGK or IGL rearrangement, expression of biallelic-rearrangements (or potential biclonality) was found in a few cell lines (Additional file 1: Figure S2). For example, two productive IGK rearrangements are detected in cell line KMS-27 (625,058 reads of IGKV2–40-IGKJ4 and 382,112 reads of IGKV1–39-IGKJ1).

**Fig. 3:**
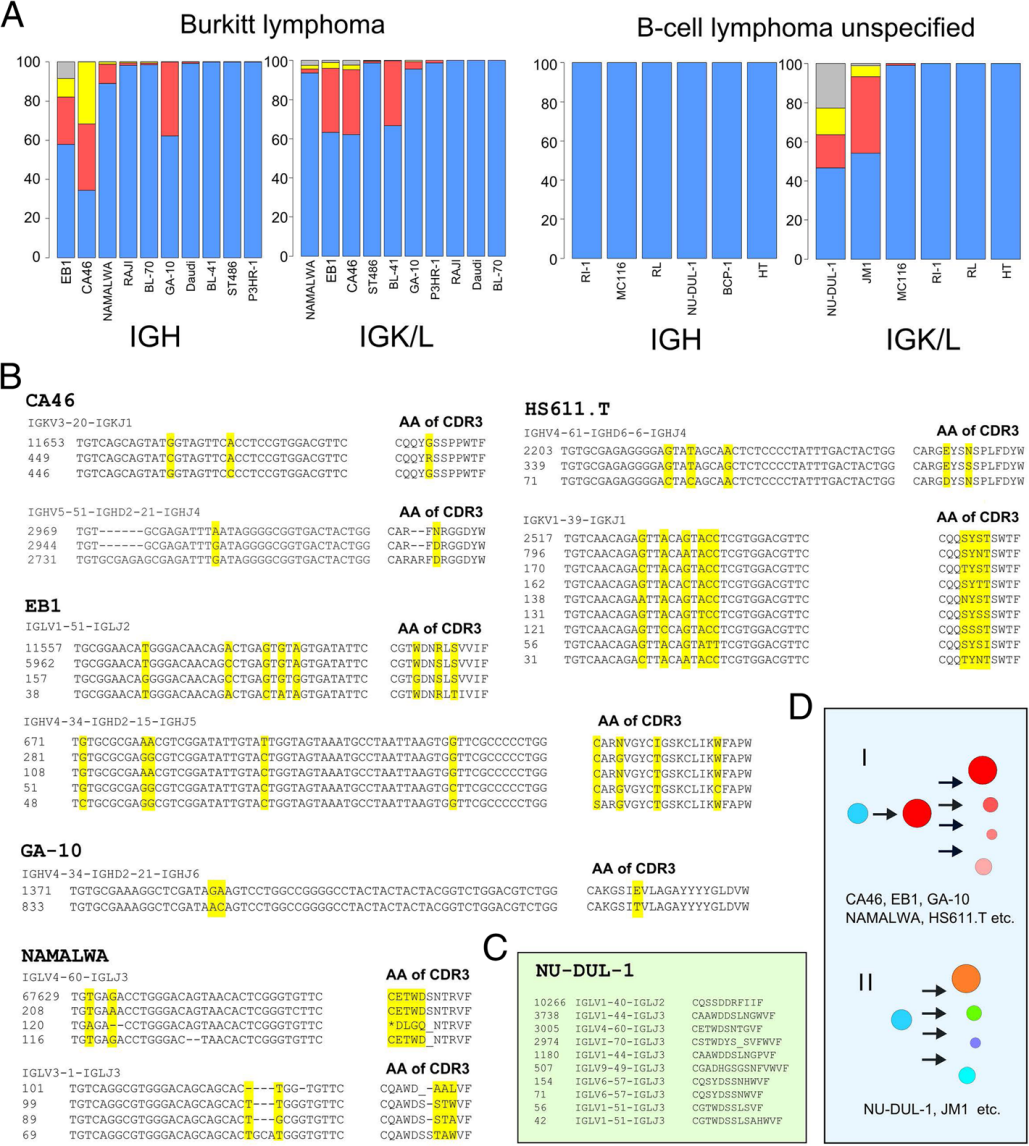
Potential subclonality and biclonality of cancer cell lines. **a** IGH and IGK/L rearranged fraction (filtered by > 30 reads) of Burkitt lymphoma and unspecified B cell lymphoma. Blue color indicates the rearrangement fraction of the dominant rearrangement, Red color indicate the rearrangement fraction of second dominant rearrangement, Yellow color indicate the third dominant rearrangement, while smaller subclonal rearrangements (≥ 4) were aggregated and labelled in grey (See also Additional file 1: Figure S2B). The subclonality of Burkitt lymphoma cell lines CA46, EB1, GA-10, NAMALWA and Hodgkin lymphomas cell line HS611.T. The RNA sequencing read counts are indicated in front of each VDJ junction nucleotide sequence. The translated CDR3 amino acids sequences are listed right after each nucleotide sequence. **c**. The potential bi/oligoclonality of unspecified B cell lymphoma cell line NU-DUL-1. Multiple IGLV rearrangements were detected in this cell line. The translated CDR3 amino acids sequences are listed right after each nucleotide sequence. **d**. Diagram shows the possible evolution path of cell lines with subclonality or biclonality. Pattern I, subclonal rearrangements derived from the dominant rearrangement through somatic hypermutations. Pattern II, multiple rearrangements evolved independently

Potential subclonality was found in several Burkitt lymphoma cell lines (e.g., NAMALWA, EB1 and CA46, Fig. 3). Subclonal rearrangements in these cell lines share the same rearrangement and similar CDR3 sequence. For example, three similar IGH rearrangements were detected in cell line CA46 (Additional file 1: Table S1). These IGH rearrangements share the identical IGH rearrangement pattern (IGHV5–51-IGHD5–12-IGHJ4) but they are slightly different in their CDR3 sequences (CARFNRGGDYW, CARFDRGGDYW, CARARFDRGGDYW, Fig. 3b), suggesting that subclonal rearrangements may have derived from dominant rearrangements through somatic hypermutations. Similarly, four IGLV4–60-IGLJ3 rearrangements were detected in the cell line NAMALWA. Three of them were barely expressed (208 reads, 120 reads and 116 reads, among which two of them are non-productive) and the sequences are highly similar to the dominant rearrangement (67,629 reads, Additional file 1: Table S1). These three minor subclonal rearrangements also appear to be derived from the dominant clone through somatic hypermuation.

Potential subclonality was also found in diffuse large B cell lymphoma (DLBCL) cell lines Pfeiffer and Toledo (Additional file 1: Table S2). All DLBCL cell lines (*n* = 17), except two (A3/KAW, CPM = 0.11, and U-937, CPM = 0.21), express high levels of clonal rearrangement of both IGH and IGL/K (Additional file 1: Table S1) [9, 10]. Indeed, U-937 is well recognized as an AML cell line instead of a DLBCL, despite the fact that this cell line was originally established from pleural effusion of a patient with histiocytic lymphoma and is still categorized as a histiocytic lymphoma by both ATCC (https://www.atcc.org/Products/All/CRL-1593.2.aspx) and CCLE. The analysis of the expression of major lineage genes also suggests that these two cell lines should not be grouped together with other DLBCL cell lines, as none of them express any typical B cell genes such as CD19, CD20 or CD79. Notably, cell line A4/Fuk also displays an abnormal expression pattern of major lineage specific genes. Conclusions of experiments reached from this cell line for DLBCL research may also need to be interpreted with caution.

Except for BCP-1 and CI-1, cell lines derived from unspecified B cell lymphoma (*n* = 4) also express high level of heavy and light chain BCR rearrangements. Cell line BCP-1 shows an inconsistent gene expression pattern as compared with other B cell lymphoma lines (e.g., no expression of CD19, CD20), suggesting that it might need to be reclassified into other blood cancer types. In contrast to the finding of subclonality in Burkitt lymphoma or DLBCL, cell lines NU-DUL-1 and JM1 contain more than 3 different IGLV rearrangements (Fig. 3c, e.g., in cell line NU-DUL-1, 10,266 reads were detected for the rearrangement IGLV1–40-IGLJ2; 3738 reads for IGLV1–44-IGLJ3; 3005 reads for IGLV4–60-IGLJ3, 2974 reads for IGLVI-70-IGLJ3, 1180 reads for IGLV1–44-IGLJ3 (encoding a different CDR3) and 507 reads for IGLV9–49-IGLJ3), suggesting a potential bi/oligoclonaity of these cell lines and that subclonal rearrangements had likely evolved independently.

Hodgkin lymphoma cell lines (*n* = 8) were derived from either mature B cells (germinal center (GC) or post GC B cells), but are also known to have a global down-regulation of B cell gene expression and a general loss of B cell phenotype [11]. Consistent with this notion, analysis of these cell lines confirmed down-regulation of B cell genes such as CD19, CD20, EBF1, etc. (except for the cell line HS.611 T, which still expresses high levels of CD19/CD20 and CD79A/B). Only two of 8 Hodgkin lymphoma cell lines express low levels of BCR rearrangements: cell line HS.611 T (IGKV1–39-IGKJ1, 2517 reads, CPM = 14.1; this cell line is EBV positive and has a high level of EBV viral gene expression) and KM-H2 (IGKV4–1-IGKJ4, 878 reads, CPM = 6.38). Notably, multiple subclonal rearrangements were detected in the cell line HS611.T, and all of them share the same IGH or IGKV rearrangement as well as highly similar CDR3 junction sequences, these subclonal rearrangements were likely derived from the major clonetypes through somatic hypermutation (Fig. 3b, d).

In contrast to the aforementioned cell lines arising from mature B cells, most of the B-ALL cell lines (*n* = 15) express low levels of IGH rearrangement as their dominant clonotypes. Consistent with the notion that most of the ALL cells had arrested in early stages of B/T cell development [12], RAG1/2 (the enzymes mediating the VDJ rearrangement process) are highly expressed in most ALL cell lines (both B and T cell ALL, Additional file 1: Figure S1). Three of the ALL cell lines had either extremely low or undetectable clonal BCR [MHH-CALL-2 (CPM = 0.06), SEM (no dominant BCR clone), REH (CPM = 0.66)]. This may suggest a lack of complete IGH and IGK/L rearrangements in immature lymphoid neoplasms, where transformation likely occurred prior to BCR rearrangement (before the pre-B stage) [13]. Transcription of biallelic-rearrangements of IGH or IGK/L or potential biclonality was found in 5 of 15 B-ALL cell lines (33.3%, RCH-ACV, 697, KOPN-8, RS4;11 and NALM-6) (Additional file 1: Figure S2 and Table S2). Intriguingly, in B-ALL cell line MHH-CALL-4, 214 reads of nonproductive TCR (TRAV8–2-TRAJ8) and 163 reads of nonproductive BCR (IGHV2–26-IGHD2–21-IGHJ5) rearrangements were detected. This cell line expressed typical B cell lineage gene such as CD19, CD79 instead of T cell lineage gene (such as CD3), suggesting cross lineage rearrangement [14, 15] or potential mixed immunophenotype of this cell line (Additional file 1: Table S2).

In general, rearrangements of IGK and IGL were highly expressed as dominant rearrangements in most B cell malignancy, highlighting the importance of examining the IGK and IGL loci in future BCR repertoire and clonality analysis. The usage of IGKV/IGLV gene segments with the highest expressed rearrangement in the B-ALL, T-ALL, Burkitt lymphoma, DLBCL and multiple myeloma cohorts is summarized in Fig. 2. Most lymphocyte derived blood cancer cell lines express high levels of a productive rearrangement, except for ALL. Almost half of all B-ALL (7 out of 15 B-ALL) cell lines carry non-productive BCR in either both alleles or in the only expressed allele (Table 1). This observation is in agreement with our recent finding that ~ 41% primary pediatric ALL samples (91 out 219 cases) lack productive BCR expression [16]. B cells carrying nonproductive BCR may bypass the BCR checkpoint through acquisition of driver mutation(s) which mimic BCR signaling (e.g., BCR-ABL1) [13, 17–24]. Alternatively, as noted recently, the pre BCR may function as a tumor suppressor in the majority of precursor B-ALL [19].

**Table 1:** Blood cancer cell lines carry nonproductive BCR/TCR in either both alleles or the only expressed allele

	Cell lines	Reads	Fraction %	V genes	D genes	J genes	CDR3 Sequences
B-ALL	NALM-19	425	36.08	IGHV1-3	IGHD6-6	IGHJ4	CARDRV*QL_PPPLRDYW
	MHH-CALL-4	214	14.33	TRAV8-2		TRAJ8	CVVGL_QKLVF
		163	10.92	IGHV2-26	IGHD2-21	IGHJ5	CARIRPRAVR_CGSP*GPDPW
	KASUMI-2	3491	60.82	IGLV1-40		IGLJ3	CQSYD_AGVF
	MUTZ-5	1632	76.30	IGLV2-34		IGLJ2	CSSYA_HLVVF
	REH	100	16.21	IGHV3-15	IGHD3-10	IGHJ6	CTTGMVRGVI_YYYYGMDVW
	RS4;11	361	26.05	IGHV6-1	IGHD1-20	IGHJ4	CAREP*LELFDYW
		241	17.39	IGHV3-20	IGHD2-8	IGHJ5	CARD*SRY*W_VCYTDWFDPW
		173	12.48	IGLV11-55		IGLJ7	CAMG_PQF
		36	2.60	IGLV4-3		IGLJ3	CGESHTIDG_SRLRFWVF
	NALM-6	15670	74.70	IGLV2-14		IGLJ7	CSSYTSS_ALGAVF
		2479	11.82	IGLV4-3		IGLJ3	CGESHTIDGQ_RLQAPGGVF
		2229	10.63	IGHV1-69	IGHD3-10	IGHJ6	CARDRRGEWPPSDYYYYYMDVW
T-ALL	KE-37	656	56.21	TRBV3-2	TRBD1	TRBJ1-5	CASSQDSGTG_RVGNQPQHF
	ALL-SIL	2364	51.42	TRGV7		TRGJ1	CATWGSG_YYKKLF
		909	19.77	TRBV18	TRBD1	TRBJ2-1	CASSPMEK_GHKGEQFF
		482	10.49	TRBV7-9	TRBD1	TRBJ2-7	CASSLDT_WYEQYF
		221	4.81	TRGV9		TRGJ1	CALWR*_YYKKLF
		86	1.87	TRDV2		TRDJ1	CACDK_DKLIF
	MOLT-3	2913	6.95	TRBV10-3		TRBJ2-5	CAISEPTG_SEETQYF
		231	0.55	TRAV1-1		TRAJ33	CAVRDHPW_SNYQLIW
		97	0.23	TRAV1-1		TRAJ24	CAVKMEQ_WGKLQF
B cell lymphoma unspecified	JM1	10946	48.32	IGLV3-1		IGLJ6	CQAWD_QPNVF
		7877	34.78	IGLV3-10		IGLJ6	CYSTDSS_VIIANVF
		1135	5.01	IGLV8-61		IGLJ7	CV_VF
	HT	18074	89.54	IGKV3-11		IGKJ5	CQQRTNWPITF
		1488	7.37	IGHV3-53	IGHD1-1	IGHJ4	CARASFAT_*LYFDSW
Blast phase CML B-ALL^#^	BV-173	764	57.83	IGHV3-21	IGHD2-15	IGHJ3	CASQIL*WW*_PYRGAFDIW
		157	11.88	IGKV2-29		IGKJ3	*MQGIH_SSLFTF
		35	2.65	TRAV8-7		TRAJ19	CAGADRLQTGMRGAF
DLBCL	Toledo	11815	58.54	IGLV2-14		IGLJ7	CSSYTS_QHSVF
		6830	33.84	IGLV3-21		IGLJ6	CQVWDSS_*SPNVF
		316	1.57	IGLV8-61		IGLJ7	CV_VF

Asterisks “*” indicate stop codon; underscores “_” indicate out of frame CDR3 translation

# BV-173 is also been regarded as an B cell precursor ALL cell lines [47] despite its original establishment from a patient with blast phase CML [48] (https://www.dsmz.de/catalogues/details/culture/ACC-20.html)

### TCR repertoire of cell lines derived from T lymphocytes

T lymphocyte cancer cell lines include T-ALL and T cell lymphoma. Remarkably, all of the five cell lines established from patients with anaplastic large cell lymphoma carry the same TCR alpha chain rearrangement (TRAV40-TRAJ4) with an identical CDR3 sequence (CLLGSISLGILSQ, 170–250 reads, Additional file 1: Table S1). The identical rearrangement/CDR3 sequence has also been detected in DEL, a cell line established from malignant histiocytosis [25] but also been recognized as an ALK-positive anaplastic large-cell lymphoma cell line [26].

Mycosis fungoides-Sezary syndrome belongs to cutaneous T-cell lymphoma (cell lines = 3). This disease is a neoplasia of T lymphocytes often possessing helper/inducer cell surface phenotype [27]. Expression of productive alpha and beta TCR rearrangements (200–5000 reads) occur in all three cell lines in the cohort. Expression of productive alpha and beta TCR rearrangements were also found in a peripheral T cell lymphoma cell line (SUP-T11) and an adult T cell lymphoma-leukemia cell line (HH) (Additional file 1: Table S2).

With the exception of the cell line ALL-SIL, which has a TCR gamma rearrangement (TRGV7-TRGJ1), the other 14 T-ALL cell lines express TCR beta rearrangements as their dominant clonotypes (TRBV-TRBD-TRBJ or TRBV-TRBJ). Most of the T-ALL cell lines express biallelic-rearrangements [28–30] or potential biclonality, containing more than one alpha or beta TCR rearrangements (Additional file 1: Table S2, Additional file 1: Figure S3). In line with allelic exclusion, the second TCR allele often encodes a non-productive CDR3 or a poorly expressed transcript (For example, in cell line MOLT-16, 15,457 reads were detected for the productive dominant rearrangement TRBV20–1-TRBD1-TRBJ2–3, while only 837 reads were detected for the putative second allele, TRBV27-TRBD1-TRBJ1–1, which codes an out of frame CDR3: CASTDPDR_EWTEAFF). Similar to B-ALL, a number of T-ALL cell lines carry non-productive rearrangements in either both alleles or in the only expressed allele, resulting in a complete lack of expression of functional TCR. For example, cell line ALL-SIL express two TCR beta rearrangements and two gamma rearrangements, and all of these rearrangements code for out of frame CDR3 (Table 1, Additional file 1: Table S2). The prevalence of non-functional TCR/BCR in ALL (both B and T ALL) again supports the recent hypothesis that the TCR/BCR might play a tumor suppressive role in most precursor ALL [19].

### Myeloid derived cancer cell lines and solid tumor cell lines

Cell lines derived from solid tumors and myeloid cells do not undergo B/TCR rearrangements. In almost all of the solid tumor cell lines (except for the few which would be discussed here) and myeloid derived cancer cell lines (AML, *n* = 31; blast phase CML, *n* = 12; CML, *n* = 2; essential thrombocythaemia, *n* = 1), less than 50–100 BCR/TCR reads were detected in the dominant rearrangement (CPM < 0.5). A few rearrangements appear to be commonly detected in a number of solid cancer and AML cell lines with very low read counts. For example, the rearrangements TRAV8–7 (non-functional segment)-TRAJ19 (non-functional segment) were commonly found in many cell lines with extremely low read count (< 30). These barely expressed rearrangements may be caused by non-specific sequencing noise or possibly due to the trace amount of cross contamination during sample preparation and sequencing [31–33].

For the 12 blast crisis CML cell lines, a few of them (CML-T1, NALM-1, BV-173) carry clonal rearrangements (Additional file 1: Table S2), indicating that these cell lines were established from “lymphoid” blast crisis [34]. For example, rearrangements of BCR heavy chain (IGHV3–9-IGHD2–21-IGHJ6, 5346 reads) were detected in NALM-1. Indeed, similar to ALL, NALM-1 expresses high levels of RAG1/2 and CD19/20/CD79A/B as well as antigens specific to ALL [35].

Remarkably, one gastric cancer cell line, KE-97, expresses high levels of IGL and IGH rearrangements (IGLV3–21-IGLJ1, 19,374 reads, IGHV1–46-IGHD3–10-IGHJ4, 2129 reads). This cell line was derived from a mucinous gastric adenocarcinoma from a 52-year-old Japanese male [36]. Considering 1–4% of the gastrointestinal malignancies are gastric lymphoma [37], this cell line was likely derived from a gastric lymphoma instead of a gastric carcinoma. Alternatively, as suggested by a previous single-nucleotide polymorphism (SNP) array analysis, the SNP pattern of this cell line was highly similar to that of KMS-18 [4], a multiple myeloma cell line established in Japan a few years after KE-97 [38]. This could represent a potential mix-up between these two cell lines.

### EBV transformed normal B lymphocyte cell lines (cohort 462 lines)

**Fig. 4:**
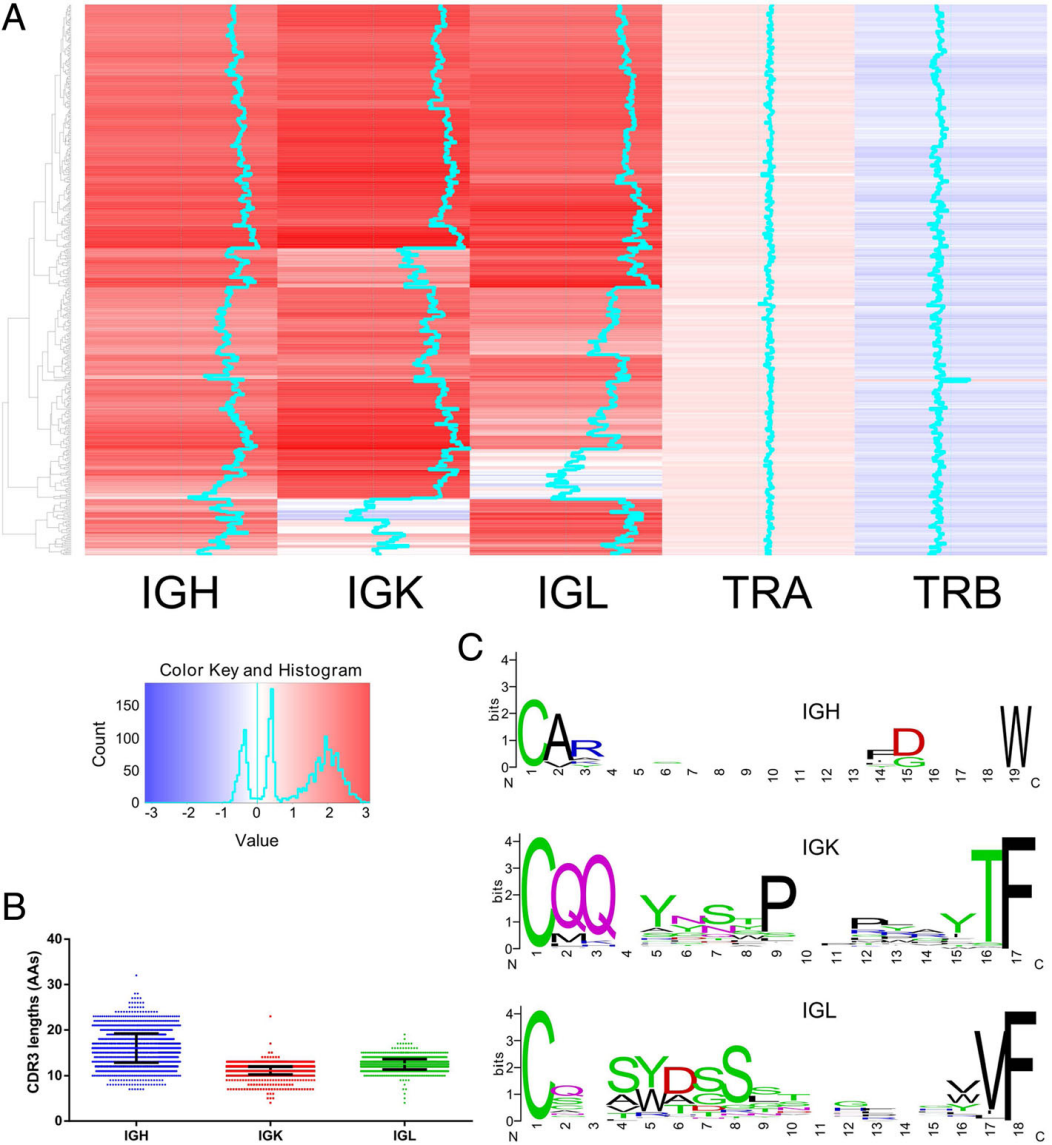
Heatmap and consensus sequence of CDR3 region of 462 EBV transformed normal B lymphocytes. **a**. Heatmap of IGH, IGK/IGL and TRA/TRB in 462 EBV transformed normal B lymphocyte cell lines. Green color line in the heatmap represents the gene expression of the particular sample, with a line drawn more towards the right, indicating a higher level of expression. **b**. Length of the CDR3 region (amino acids sequence, AAs) of the dominant clone in 462 EBV transformed normal B lymphocytes. **c**. Consensus sequence of the CDR3 region of the dominant clone in 462 EBV transformed normal B lymphocyte cell lines. Sequences of the CDR3 region were aligned using ClustalW, and the consensus sequences were plotted using weblogo

**Fig. 5:**
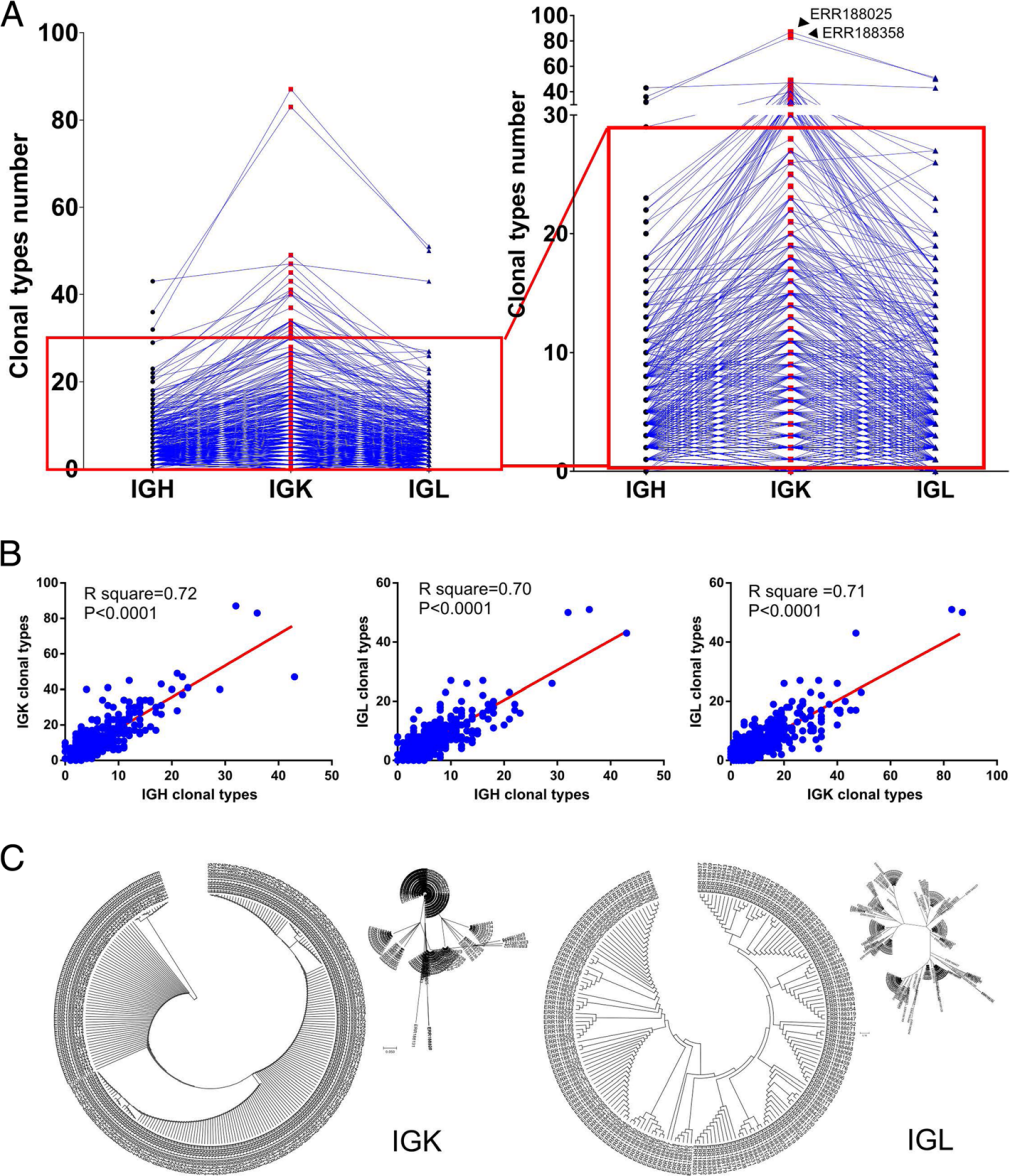
Clonotypes of IGH, IGK/L in 462 samples of EBV transformed normal B lymphocytes. **a**. Number of clonotypes based on IGH vs IGK; IGH vs IGL; and IGK vs IGL in 462 EBV transformed normal B lymphocyte lines. Right panel is the blow-up of the picture (left panel, clonal type 0–30). **b**. Correlation of the number of clonal types based on IGH vs IGK; IGH vs IGL; and IGK vs IGL in 462 samples of EBV transformed normal B lymphocytes. **c**. Phylogenetic trees inferred based on CDR3 region of either IGK or IGL rearrangements of the dominant clone of 462 EBV transformed normal B lymphocyte cell lines, using Neighbor-Joining method conducted with MEGA7. Evolutionary distances are computed using the Poisson correction method and are in the units of the number of amino acid substitutions per site

The same bioinformatics pipeline was used to analyze 462 EBV transformed normal B lymphocyte cell lines which were immortalized from healthy donor’s B cells. Rearrangements of both IGH and IGK/L were readily detected in almost all of the lymphocyte cell lines (Fig. 4a). IGH rearrangements were found in 459 cell lines, while IGK and IGL rearrangements were detected in 426 and 421 cell lines, respectively, supporting a notion that most of the EBV immortalized cells were derived from mature B cells. In contrast to the highly diversified repertoire of normal circulating B cell population, clonality occurred in the majority of EBV transformed B lymphocyte cell lines, reflecting a loss of diversity and clonal evolution during the establishment, growth and subculture of these cell lines. Among the 462 B-lymphocyte cell lines, 54 appeared to be monoclonal as only one IGH or IGK/IGL rearrangement is detected (with comparable sequencing depth of other cell lines). Potential biallelic rearrangement or biclonality (2 IGH or IGL or 2–4 IGK) was detected in the other 53 cell lines. For the other 355 cell lines, bi/oligo or polyclonality (> 2 IGH or > 2 IGL or > 4 IGK [7]) was detected. Most of these have less than 20 clonal rearrangements (with > 100 reads threshold) and many of them have one highly expressed dominant rearrangement with high clonotype faction (Fig. 5a, Additional file 1: Figure S4). The clonal status of different loci appears correlated and cell lines with polyclonality of one locus tend to display polyclonality at the other loci (Fig. 5a, b). A few cell lines still retained a relatively diversified population. For example, the cell lines ERR188358 and ERR188025 contain more than 80 different IGK (83 and 85, respectively) and more than 30 different IGH rearrangements (Fig. 5a). The inferred phylogenetic trees based on the CDR3 region of the dominant rearrangements of these 462 lymphocyte lines are shown in Fig. 5c.

**Fig. 6:**
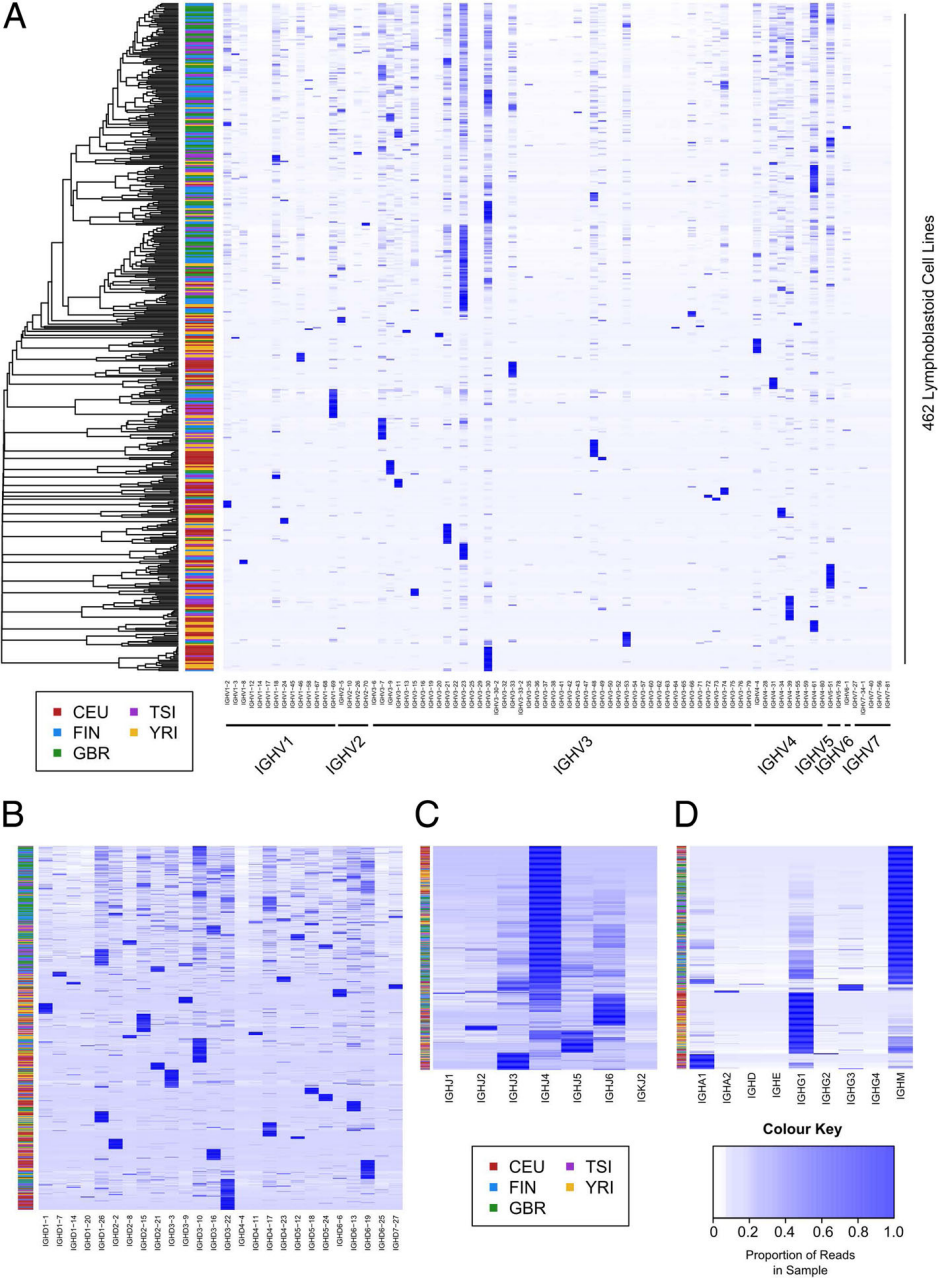
Heatmap depicting the gene segments usage of IGHV (**a**), IGHD (**b**), IGHJ (**c**) and the constant region (**d**) of 462 EBV transformed normal B lymphocyte cell lines. These cell lines were derived from five populations: Utah Residents (CEPH) with Northern and Western European Ancestry (CEU), Finnish in Finland (FIN), British (GBR), Toscani in Italia (TSI) and Yoruba in Ibadan, Nigeria (YRI) [5]

We hypothesize that EBV mediated transformation occurred in a number of B cells, and during culture, some clones outcompeted the others and gradually became dominant clones. Therefore, diversity was lost and the population became oligoclonal or even monoclonal [39, 40]. In total, 9827 different rearrangements of IGH/IGK/IGL (with a cut off of at least 100 sequencing reads, here after referred as index rearrangement) were detected in 426 EBV transformed B lymphocytes lines; 8 of them are highly expressed as prominent rearrangements which occupies more than 90% of the clonotype fraction in their corresponding cell lines (Additional file 1: Table S3). All (except two) of the dominant rearrangements of the 462 B lymphocyte lines encode a productive BCR. Among the 9827 index rearrangements detected in these cell lines, only 363 subclonal rearrangements codes for nonproductive BCR (353 out of frame, 50 interrupted by inside stop codon, 40 have both inside stop codon and out of frame rearrangements). The majority of these non-productive BCR rearrangements were barely expressed. Among all the 462 lymphocyte cell lines, only one TCR rearrangement was detected (TRBV7–9-TRBJ2–1, 143 reads) in cell line ERR188361 (Additional file 1: Table S4). To gain further insight into the BCR repertoire of EBV transformed normal B lymphocyte, we generated a heatmap of heavy/light chain gene segment usage of these 462 lymphocyte lines (Fig. 6, Additional file 1: Figure S4). Analysis of the IGH gene usage revealed a modest, potential population bias of the IGHV segments usage (for example, increase of IGHV3–23 usage was noted in cell lines established from Finnish (FIN) and British (GBR) individuals, Fig. 6a), which may reflect an exposure history of certain common antigens in each population [41–43].

## Discussion

One of the potential pitfalls of profiling the BCR/TCR repertoire using RNA sequencing is that non-transcribed rearrangements may not be detected, and the clonality analysis may be biased by the expression level of BCR/TCR loci. Nevertheless, in comparison to traditional PCR based method which is labor intensive and requires well trained staffs, NGS/RNA sequencing based analysis is more standardized and can be simply outsourced to big sequencing centers. In contrast to PCR based analysis which is limited by the number of loci that can be feasibly examined, NGS based profiling can comprehensively detect all expressed TCR/BCR rearrangements, including some rearrangements which are not usually included in PCR based analysis (e.g., TCRA alpha rearrangement). The BCR/TCR repertoire identified using RNA sequencing were consistent with previous studies using BIOMED-2 PCR Sanger sequencing [29, 30] (The TCR gene names in their paper utilized old aliases and need to be converted to standard gene name using Genecards https://www.genecards.org). In addition, the feasibility of profiling BCR repertoires from RNA sequencing data have recently been explored in CLL [44]. In their study comparing two approaches, BCR repertoire profiling using RNA sequencing showed equal or superior results as compared with traditional PCR and Sanger sequencing (clinical technique) [44]. In addition, with a few exceptions which are likely caused by accidental mix-up, a consistent BCR/TCR repertoire could be obtained when comparing the RNA sequencing data from CCLE with sequencing results for blood cancer cell lines generated by different researchers (for example, cancer cell lines RNA sequencing data in SRA database, https://www.ncbi.nlm.nih.gov/sra). This suggests that the BCR/TCR repertoire pattern may be used as an alternative/complement authorization method for lymphocyte derived cell lines. The advantage of this approach is that when RNA sequencing data is available (which is already widely applied in many kinds of research), the cell lines (need to be lymphocyte derived cells) used in these studies can be simultaneously validated based on the BCR/TCR profile. As such, the RNA sequencing data deposited in the online public database (GEO, SRA) can be further checked and authenticated independently by any researchers. Crucially, we noticed that poly A selection before sequencing is important for examining the BCR/TCR repertoire, as poly A enriched samples generated 100-fold more BCR/TCR sequencing reads as compared to samples which were not subjected to  poly A selection but were sequenced at similar sequencing depth. Nonetheless, RNA sequencing of samples without poly A selection can still discover the majority of BCR/TCR rearrangements. However, accuracy of clonotype fraction of subclonal rearrangements may be significantly affected.

Currently, multiplex PCR based T/B cell receptor rearrangement testing has been used as a clinical approach to detect suspected lymphoproliferative disease [45]. This has occasionally been hindered by a deletion/translocation (e.g., t(11;14)) event in the Ig loci and different set of primer panels may be tested before clonality can be inferred. Recently, the cost of next-generation sequencing (NGS) has quickly decreased and gradually become comparable with traditional PCR-Sanger analysis. RNA sequencing is straightforward with standardized procedures, eliminating the need of patient based personalized BCR/TCR primer sets selection and optimization. Furthermore, RNA sequencing can be scaled up to a large number of samples easily, allowing simultaneous examination of gene expression, SNP and somatic mutations, in addition to the B/TCR rearrangement repertoire. Our analysis highlights the potential of using RNA sequencing as a diagnostic test to examine the BCR/TCR clonal rearrangement in lymphoid malignancy.

The observation of subclonality or potential biclonality in a number of blood cancer cell lines is interesting. In most of these cases, the subclones appear to have been derived from the major clones through somatic hypermutation (e.g., Burkitt lymphoma cell lines NAMALWA, GA-10, EB1, CA46 and Hodgkin lymphoma cell line HS611.T etc.). However, in the B cell lymphoma cell lines NU-DUL-1, JM1 and the Multiple myeloma cell line AMO-1, more than three different IGL (cell lines NU-DUL-1, JM1) or IGH (cell lines AMO-1) rearrangements were detected, suggesting independent biclonality in these cell lines. As cancer cell lines are generally regarded as of monoclonal origin, the potential biclonality at these cell lines is interesting and may require further detailed study. On the other hand, our observation of high clonality and potential clonal selection/evolution in EBV transformed normal B lymphocytes suggests that careful experimental design and interpretation of the result may be required when using EBV transformed lines as a model to study normal B cell population, B cell gene expression or quantitative trait loci (QTL) [40, 46].

## Conclusions

In summary, we comprehensively profiled the B/T cell receptor repertoire in 936 cancer cell lines and 462 samples of EBV transformed normal B lymphocytes. The relative “pure” feature of cancer cell lines circumvents the problem of tumor infiltrating T/B and stroma cells in primary tumor samples. Our analysis provides unique insights into the BCR/TCR rearrangement repertoire and clonality of cell lines derived from lymphocyte cells.

## Additional files

Additional file 1: **Figure S1**. Expression of CD19/CD20, CD79A/B and RAG1/RAG2 in blood cancer cell lines. X axis: 164 blood cancer cell lines grouped base on the disease types (rectangle color bar below the X axis), each dot in X axis represent one cell line, and the Y axis, expression level (log2 value) of indicated genes. **Figure S2**. Clonal fraction (filtered by >30 reads) of B-ALL, multiple myeloma, diffuse large B cell lymphoma, Burkitt lymphoma, B cell lymphoma (unspecified) and mantle cell lymphoma base on IGH or IGHK/L. Blue color indicates the clonotype fraction of the most dominant clone, Red color indicate the clonotype fraction of second dominant clone, Yellow color indicate the third dominant clone, while any smaller subclones were aggregated and labelled in grey. **Figure S3**. Clonal fraction (filtered by >30 reads) of T-ALL and anaplastic large cell lymphoma base on TRCA or TRCB. Blue color indicates the clonotype fraction of dominant clone, red color inidicate the clonotype fraction of second dominant clone, while gray color indicate the third dominant clone. **Figure S4**. Heatmap showing the usage of IGK/L V genes (A), IGK/L J genes (B), and constant region (C) in 462 samples of EBV transformed normal B lymphocytes. **Figure S5**. The phylogenetic tree inferred based on the rearrangement of the CDR3 region of IGH, IGK and IGL of EBV transformed B lymphocyte samples ERR188025, ERR188358 and ERR188212. These three cell lines have much higher number of rearrangement types than the other B lymphocyte lines. Clonal Fraction (upper panel) and the read counts (lower panel) of the dominant clone of 462 samples of EBV transformed normal B lymphocytes. (ZIP 1290 kb)

## Abbreviations

B/TCRs: B/T cell receptors
CCLE: Cancer Cell Line Encyclopedia
EBV: Epstein-Barr Virus
V(D)J: variable (V), diversity (D) and joining (J)
