# Supplementary material for: Profiling the B/T cell receptor repertoire of lymphocyte derived cell lines
Source: BMC Cancer. 2018 Oct 1;18:940. doi: 10.1186/s12885-018-4840-5 (PMC6167786; doi:10.1186/s12885-018-4840-5)
Supplement: Supplementary file 1 — Figure S1. Expression of CD19/CD20, CD79A/B and RAG1/RAG2 in blood cancer cell lines. X axis: 164 blood cancer cell lines grouped base on the disease types (rectangle color bar below the X axis), each dot in X axis represent one cell line, and the Y axis, expression level (log2 value) of indicated genes. Figure S2. Clonal fraction (filtered by >30 reads) of B-ALL, multiple myeloma, diffuse large B cell lymphoma, Burkitt lymphoma, B cell lymphoma (unspecified) and mantle cell lymphoma base on IGH or IGHK/L. Blue color indicates the clonotype fraction of the most dominant clone, Red color indicate the clonotype fraction of second dominant clone, Yellow color indicate the third dominant clone, while any smaller subclones were aggregated and labelled in grey. Figure S3. Clonal fraction (filtered by >30 reads) of T-ALL and anaplastic large cell lymphoma base on TRCA or TRCB. Blue color indicates the clonotype fraction of dominant clone, red color inidicate the clonotype fraction of second dominant clone, while gray color indicate the third dominant clone. Figure S4. Heatmap showing the usage of IGK/L V genes (A), IGK/L J genes (B), and constant region (C) in 462 samples of EBV transformed normal B lymphocytes. Figure S5. The phylogenetic tree inferred based on the rearrangement of the CDR3 region of IGH, IGK and IGL of EBV transformed B lymphocyte samples ERR188025, ERR188358 and ERR188212. These three cell lines have much higher number of rearrangement types than the other B lymphocyte lines. Clonal Fraction (upper panel) and the read counts (lower panel) of the dominant clone of 462 samples of EBV transformed normal B lymphocytes. (ZIP 1290 kb) [file 12885_2018_4840_MOESM1_ESM.zip › Supplementary data 20170908R2.docx]

**Supplementary Figure legends**

**Supplementary Figure 1. Expression of CD19/CD20, CD79A/B and RAG1/RAG2 in blood cancer cell lines.** X axis: 164 blood cancer cell lines grouped base on the disease types (rectangle color bar below the X axis), each dot in X axis represent one cell line. The Y axis shows expression level (log2 value) of indicated genes.

**Supplementary Figure 2. Rearrangement fraction (filtered by >30 reads) of B-ALL, multiple myeloma, diffuse large B cell lymphoma, Burkitt lymphoma, B cell lymphoma (unspecified) and mantle cell lymphoma base on IGH or IGHK/L.** Blue color indicates the rearrangement fraction of dominant rearrangement, Red color indicate the rearrangement fraction of second dominant rearrangement, Yellow color indicate the third dominant rearrangement, while any smaller subclonal rearrangements (≥ 3) are aggregated and labelled in grey.

**Supplementary Figure 3. Rearrangement fraction (filtered by >30 reads) of T-ALL and anaplastic large cell lymphoma base on TRCA or TRCB.** Blue color indicates the rearrangement fraction of dominant rearrangement, Red color indicates the rearrangement fraction of second dominant rearrangement, Yellow color indicates the third dominant rearrangement, while any smaller subclonal rearrangements (≥ 3) are aggregated and labelled in grey.

**Supplementary Figure 4. Heatmap shows the usage of IGK/L V genes (A), IGK/L J genes (B), and constant region (C) in 462 samples of EBV transformed normal B lymphocytes.** These cell lines are derived from five populations: Utah Residents (CEPH) with Northern and Western European Ancestry (CEU), Finnish in Finland (FIN), British (GBR), Toscani in Italia (TSI) and Yoruba in Ibadan, Nigeria (YRI).

**Supplementary Figure 5. Fraction (upper panel) and the read counts (lower panel) of the dominant rearrangement of 462 samples of EBV transformed normal B lymphocytes.** X axis: 462 samples of EBV transformed normal B lymphocytes. Y axis: rearrangement fraction (upper panel) and the read counts (lower panel) of the dominant rearrangement of 462 samples of EBV transformed normal B lymphocytes.

**Additional file 1: Table S1. Dominant (highest expressed) rearrangement of 164 blood cancer cell lines.**

| Disease | Cell line | All V hits | All D hits | All J hits | Read counts | Clonotype Fraction % | CPM |
| --- | --- | --- | --- | --- | --- | --- | --- |
| B-ALL | 697 | IGHV2-26(301.6) | IGHD2-2(120) | IGHJ4(157.8) | 1373 | 65.38 | 6.94 |
|  | BDCM | IGLV4-60(399.1) | | IGLJ2(141.6),  IGLJ3(117.6) | 32303 | 69.88 | 168.53 |
|  | KASUMI-2 | IGLV1-40(444.1) | | IGLJ3(155.9) | 3491 | 60.82 | 29.14 |
|  | KOPN-8 | IGHV6-1(364.4) | IGHD1-1(46) | IGHJ4(195.6) | 6012 | 75.41 | 43.92 |
|  | MHH-CALL-2 | TRAV21(55.9) | | TRAJ27(64.9) | 15 | 2.88 | 0.06 |
|  | MHH-CALL-3 | IGHV3-15(248.6) | IGHD3-3(50) | IGHJ5(191.6) | 1044 | 48.11 | 5.22 |
|  | MHH-CALL-4 | TRAV8-2(395.5) | | TRAJ8(223.6) | 214 | 14.33 | 1.38 |
|  | MUTZ-5 | IGLV2-34(350.5) | | IGLJ2(163.4) | 1632 | 76.30 | 7.60 |
|  | NALM-19 | IGHV1-3(252.4) | IGHD6-6(80) | IGHJ4(160.6) | 425 | 36.08 | 1.78 |
|  | NALM-6 | IGLV2-14(382.1) | | IGLJ7(134.6) | 15670 | 74.70 | 96.56 |
|  | RCH-ACV | IGHV1-3(218.6) | IGHD5-18(71),  IGHD5-5(71) | IGHJ4(158),  IGHJ5(151.2) | 10265 | 77.69 | 41.16 |
|  | Reh | IGHV3-15(347.8) | IGHD3-10(70) | IGHJ6(185.4) | 100 | 16.21 | 0.66 |
|  | RS4;11 | IGHV6-1(340.8) | IGHD1-20(45),  IGHD1-7(45) | IGHJ4(184.4) | 361 | 26.05 | 2.33 |
|  | SEM | TRAV8-7(68.6) | | TRAJ19(54.7) | 32 | 6.53 | 0.21 |
|  | SUP-B15 | IGHV3-53(321.7) | IGHD2-8(90) | IGHJ6(157.3) | 2739 | 71.74 | 14.13 |
| T-ALL | ALL-SIL | TRGV7(395.9) | | TRGJ1(192.1),  TRGJ2(192.1) | 2364 | 51.42 | 14.35 |
|  | DND-41 | TRBV18(439.2) | TRBD1(50) | TRBJ1-2(172.7) | 4868 | 84.46 | 52.39 |
|  | HPB-ALL | TRBV5-5(379.4) | | TRBJ2-5(172.3) | 23926 | 47.87 | 112.87 |
|  | JURKAT | TRBV12-3(429.5),  TRBV12-4(403.8) | TRBD1(25) | TRBJ1-2(203.4) | 10591 | 86.02 | 76.92 |
|  | KE-37 | TRBV3-2(375.5) | TRBD1(60) | TRBJ1-5(199.5) | 656 | 56.21 | 4.44 |
|  | Loucy | TRBV5-6(334.5) | TRBD1(35) | TRBJ2-1(209.9) | 12952 | 60.90 | 102.43 |
|  | MOLT-13 | TRBV10-1(322.3) | TRBD2(28),  TRBD1(25) | TRBJ1-1(203.1) | 1345 | 58.20 | 5.92 |
|  | MOLT-16 | TRBV20-1(349.6) | TRBD1(35) | TRBJ2-3(200.9) | 15457 | 50.22 | 78.58 |
|  | MOLT-3 | TRBV20-1(404.8) | | TRBJ2-1(183) | 38063 | 90.81 | 165.78 |
|  | P12-ICHIKAWA | TRBV6-5(392.3) | | TRBJ2-1(198.2) | 14632 | 77.19 | 61.04 |
|  | PEER | TRBV4-2(326.7) | | TRBJ2-3(186.9) | 21624 | 89.06 | 104.52 |
|  | PF-382 | TRBV20-1(391.2) | TRBD2(65) | TRBJ2-1(178.3) | 16814 | 90.26 | 89.02 |
|  | RPMI-8402 | TRBV15(266.9) | TRBD1(26),  TRBD2(25) | TRBJ1-5(230.1) | 15614 | 93.11 | 81.12 |
|  | SUP-T1 | TRBV9(416.8) | | TRBJ2-1(152.4) | 12869 | 93.19 | 75.97 |
|  | TALL-1 | TRBV20-1(399.8) | | TRBJ2-5(186.1) | 9397 | 82.88 | 66.19 |
| AML | AML-193 | IGKV1-5(370.7) | | IGKJ2(124.4) | 71 | 13.79 | 0.49 |
|  | CMK | TRAV9-1(53.5) | | TRAJ26(62.3) | 18 | 4.56 | 0.09 |
|  | EOL-1 | TRAV8-7(71.2) | | TRAJ19(55) | 18 | 4.05 | 0.07 |
|  | F-36P | IGLV3-21(382.1) | | IGLJ1(144.7) | 46 | 7.28 | 0.25 |
|  | GDM-1 | TRAV8-7(67) | | TRAJ19(55) | 12 | 3.22 | 0.06 |
|  | HEL | IGLV4-60(75) | | IGLJ1(45) | 13 | 2.61 | 0.08 |
|  | HEL 92.1.7 | IGLV4-60(76.7) | | IGLJ1(44.8) | 30 | 3.84 | 0.16 |
|  | HL-60 | IGKV1-5(405.3) | | IGKJ2(122.3) | 24 | 5.15 | 0.15 |
|  | KASUMI-1 | TRAV8-7(69) | | TRAJ19(54.7) | 63 | 10.08 | 0.36 |
|  | Kasumi-6 | IGKV2-40(387.3) | | IGKJ4(148.6) | 59 | 11.07 | 0.40 |
|  | KG-1 | IGKV2-40(328.5) | | IGKJ4(153.8) | 27 | 5.41 | 0.19 |
|  | M-07e | TRAV8-7(67) | | TRAJ19(55) | 26 | 5.26 | 0.18 |
|  | ME-1 | TRAV8-7(67.9) | | TRAJ19(55) | 54 | 12.53 | 0.40 |
|  | MOLM-13 | TRAV8-7(67) | | TRAJ19(55.1) | 21 | 5.21 | 0.15 |
|  | MOLM-16 | TRAV8-7(67) | | TRAJ19(55) | 29 | 5.26 | 0.13 |
|  | MONO-MAC-1 | TRAV8-7(67) | | TRAJ19(55) | 21 | 4.32 | 0.13 |
|  | MONO-MAC-6 | TRAV8-7(67) | | TRAJ19(55) | 24 | 4.10 | 0.12 |
|  | MUTZ-3 | IGLV2-11(150.4) | | IGLJ6(49.6) | 13 | 4.38 | 0.12 |
|  | MV-4-11 | IGLV4-60(75) | | IGLJ1(45.3) | 20 | 3.21 | 0.10 |
|  | NB-4 | IGKV1-5(417.7) | | IGKJ2(120.5) | 42 | 7.58 | 0.21 |
|  | NOMO-1 | IGKV1-5(354) | | IGKJ2(130.8) | 29 | 5.19 | 0.13 |
|  | OCI-AML2 | TRAV8-7(67) | | TRAJ19(55) | 27 | 4.63 | 0.15 |
|  | OCI-AML3 | TRAV8-7(68.8) | | TRAJ19(55) | 27 | 5.82 | 0.17 |
|  | OCI-AML5 | IGKV1-39(266.1) | | IGKJ1(142.8) | 30 | 5.46 | 0.17 |
|  | OCI-M1 | TRAV8-7(65.2) | | TRAJ19(55) | 37 | 7.69 | 0.24 |
|  | P31/FUJ | TRGV10(416.6) | | TRGJP2(259.7) | 145 | 20.60 | 0.72 |
|  | PL-21 | TRAV8-7(67) | | TRAJ19(55) | 44 | 7.80 | 0.20 |
|  | SIG-M5 | TRAV8-7(68.5) | | TRAJ19(53.1) | 29 | 6.20 | 0.16 |
|  | SKM-1 | TRAV8-7(68.5) | | TRAJ19(55) | 34 | 6.80 | 0.16 |
|  | TF-1 | TRAV8-7(69.1) | | TRAJ19(54.9) | 70 | 9.42 | 0.34 |
|  | THP-1 | IGKV1-39(269.3) | | IGKJ1(151) | 29 | 5.42 | 0.18 |
| Adult T cell lymphoma-leukaemia | HH | TRBV2(301.6) | TRBD2(25) | TRBJ2-3(213.2) | 607 | 40.25 | 3.23 |
| Anaplastic large cell lymphoma | KARPAS-299 | TRBV20-1(397.8) | TRBD2(31) | TRBJ2-7(170) | 723 | 55.02 | 5.70 |
|  | Ki-JK | TRAV40(82.4) | | TRAJ4(50.9) | 210 | 29.66 | 1.40 |
|  | SR-786 | TRAV40(82.5) | | TRAJ4(49.8) | 176 | 15.20 | 0.71 |
|  | SU-DHL-1 | TRAV40(82) | | TRAJ4(50.5) | 185 | 18.39 | 0.97 |
|  | SUP-M2 | TRAV40(82) | | TRAJ4(50.6) | 224 | 21.75 | 1.36 |
| B cell lymphoma unspecified | BCP-1 | IGHV3-23(303.7) | IGHD3-10(56) | IGHJ4(187.5) | 36 | 7.76 | 0.15 |
|  | CI-1 | TRAV8-7(67) | | TRAJ19(55.2) | 25 | 4.97 | 0.11 |
|  | HT | IGKV3-11(408.4) | | IGKJ5(155.6) | 18074 | 89.54 | 91.28 |
|  | JM1 | IGLV3-1(426) | | IGLJ6(155.9) | 10946 | 48.32 | 42.32 |
|  | MC116 | IGLV2-14(408.8) | | IGLJ2(146.5) | 29091 | 66.03 | 208.60 |
|  | NU-DUL-1 | IGLV1-40(224.9) | | IGLJ2(100.5) | 10266 | 40.59 | 58.47 |
|  | RI-1 | IGKV4-1(428.1) | | IGKJ2(148.8) | 104681 | 81.36 | 474.91 |
|  | RL | IGLV2-11(228.2) | | IGLJ1(118.5) | 12391 | 72.33 | 82.06 |
| Blast phase chronic myeloid leukaemia | BV-173 | IGHV3-21(289.7) | IGHD2-15(105) | IGHJ3(181.4) | 764 | 57.83 | 3.89 |
|  | CML-T1 | TRBV19(336.7) | TRBD2(61) | TRBJ2-5(188.4) | 3251 | 62.36 | 14.74 |
|  | EM-2 | TRAV1-2(73.7) | | TRAJ9(35.3) | 12 | 2.91 | 0.05 |
|  | JK-1 | TRAV8-7(68.8) | | TRAJ19(55) | 26 | 6.79 | 0.20 |
|  | JURL-MK1 | TRAV8-7(67) | | TRAJ19(55) | 25 | 5.69 | 0.19 |
|  | K-562 | TRAV8-7(71.9) | | TRAJ19(54.9) | 46 | 7.44 | 0.27 |
|  | KCL-22 | IGKV2-40(397.6) | | IGKJ4(150.1) | 158 | 22.25 | 1.16 |
|  | KYO-1 | TRAV8-7(67.7) | | TRAJ19(55) | 71 | 14.49 | 0.56 |
|  | LAMA-84 | TRAV8-7(67) | | TRAJ19(55) | 24 | 5.00 | 0.14 |
|  | MEG-01 | TRAV8-7(67) | | TRAJ19(55) | 26 | 5.53 | 0.17 |
|  | MOLM-6 | TRAV8-7(67) | | TRAJ19(55) | 41 | 7.52 | 0.19 |
|  | NALM-1 | IGHV3-9(297.6) | IGHD2-21(95) | IGHJ6(146) | 5346 | 88.35 | 25.50 |
| Burkitt lymphoma | BL-41 | IGKV3-20(413.8) | | IGKJ2(159) | 34963 | 56.47 | 173.59 |
|  | BL-70 | IGKV1-39(475.4) | | IGKJ4(172.8) | 21725 | 69.76 | 101.41 |
|  | CA46 | IGKV3-20(493) | | IGKJ1(141) | 11653 | 41.80 | 54.19 |
|  | Daudi | IGKV1-39(333) | | IGKJ4(130) | 30696 | 70.29 | 117.43 |
|  | EB1 | IGLV1-51(309.4) | | IGLJ2(141.7) | 11557 | 57.29 | 56.98 |
|  | GA-10 | IGKV2-28(429.9) | | IGKJ2(173.6) | 28987 | 87.40 | 110.04 |
|  | NAMALWA | IGLV4-60(453.4) | | IGLJ3(140.4) | 67629 | 85.38 | 291.19 |
|  | P3HR-1 | IGKV3-15(299) | | IGKJ4(113.5) | 2373 | 74.39 | 12.03 |
|  | Raji | IGKV3-20(374) | | IGKJ2(138.3) | 38181 | 85.01 | 170.56 |
|  | ST486 | IGKV2-28(478.2) | | IGKJ2(124.4) | 18336 | 85.15 | 76.16 |
| Chronic lymphocytic leukaemia-small lymphocytic lymphoma | EHEB | IGKV1-33(369.8) | | IGKJ2(123.5) | 83657 | 79.94 | 399.89 |
|  | JVM-2 | IGLV2-14(396) | | IGLJ2(156.9) | 27509 | 63.68 | 224.67 |
|  | JVM-3 | IGKV1-5(448) | | IGKJ1(161.4) | 104390 | 67.34 | 709.06 |
|  | MEC-1 | IGKV4-1(462.6) | | IGKJ2(155.5) | 22494 | 71.17 | 132.97 |
| Chronic myeloid leukaemia | KU812 | TRAV8-7(68.8) | | TRAJ19(53) | 28 | 5.80 | 0.20 |
|  | NCO2 | TRAV8-7(67) | | TRAJ19(55) | 30 | 5.20 | 0.12 |
| Diffuse large B cell lymphoma | A3/KAW | IGKV1-39(227.6) | | IGKJ1(137.3) | 21 | 4.23 | 0.11 |
|  | A4/Fuk | IGKV1-27(394.7) | | IGKJ4(132) | 35456 | 70.78 | 233.19 |
|  | DB | IGLV2-8(139.6) | | IGLJ2(65.9),  IGLJ3(65.9) | 3521 | 52.19 | 20.24 |
|  | DOHH-2 | IGLV2-11(314.9) | | IGLJ3(136.3),  IGLJ2(131.3) | 12436 | 67.43 | 53.15 |
|  | KARPAS-422 | IGKV2-28(427.8) | | IGKJ4(143.9) | 17537 | 96.07 | 130.21 |
|  | NU-DHL-1 | IGLV3-1(403.7) | | IGLJ1(153.6) | 17821 | 41.47 | 81.93 |
|  | OCI-LY-19 | IGLV1-40(476.6) | | IGLJ3(154.5) | 6917 | 80.76 | 34.21 |
|  | OCI-LY3 | IGLV3-21(283.7) | | IGLJ3(129.5) | 10491 | 88.26 | 28.80 |
|  | Pfeiffer | IGKV1-5(368.7) | | IGKJ2(155.9) | 74652 | 89.56 | 323.49 |
|  | SU-DHL-10 | IGHV7-81(75) | IGHD6-13(30),  IGHD6-19(30),  IGHD6-6(30) | IGHJ2(150) | 4565 | 49.82 | 25.69 |
|  | SU-DHL-4 | IGKV2-28(411.3) | | IGKJ4(152.1) | 15597 | 75.84 | 82.71 |
|  | SU-DHL-5 | IGHV1-8(171.4),  IGHV1-2(164.7) | IGHD2-21(36),  IGHD2-15(35),  IGHD3-3(35) | IGHJ4(142.2) | 1779 | 50.63 | 17.74 |
|  | SU-DHL-6 | IGKV1-5(394.1) | | IGKJ1(122.7) | 19719 | 80.55 | 111.57 |
|  | SU-DHL-8 | IGLV1-44(453.3),  IGLV1-47(367.2) | | IGLJ3(151.2) | 50490 | 68.64 | 238.34 |
|  | Toledo | IGLV2-14(392.6) | | IGLJ7(143.8) | 11815 | 58.54 | 50.05 |
|  | U-937^#^ | TRAV8-7(68.2) | | TRAJ19(55.1) | 36 | 6.23 | 0.21 |
|  | WSU-DLCL2 | IGLV2-14(157.2),  IGLV2-23(131.8) | | IGLJ2(97.6),  IGLJ3(97.6) | 5825 | 70.11 | 58.82 |
| Essential thrombocythaemia | Set-2 | TRAV9-1(60.7) | | TRAJ26(66.5) | 18 | 3.03 | 0.11 |
| Hodgkin lymphoma | HD-MY-Z | TRAV8-7(67) | | TRAJ19(55) | 36 | 5.12 | 0.18 |
|  | Hs 611.T | IGKV1-39(449.9) | | IGKJ1(163.1) | 2517 | 26.69 | 14.08 |
|  | Hs 616.T | TRAV22(67) | | TRAJ40(58.1) | 24 | 4.90 | 0.14 |
|  | KM-H2 | IGKV4-1(288.6) | | IGKJ4(149.3) | 878 | 61.23 | 6.39 |
|  | L-1236 | TRAV8-7(67) | | TRAJ19(55) | 34 | 5.61 | 0.22 |
|  | L-428 | IGKV2-40(421.9) | | IGKJ4(147.5) | 33 | 6.99 | 0.22 |
|  | L-540 | TRAV8-4(297.4) | | TRAJ9(232) | 56 | 9.91 | 0.46 |
|  | TO 175.T | IGKV3-20(274.2) | | IGKJ4(115.3) | 47 | 9.46 | 0.30 |
| Mantle cell lymphoma | GRANTA-519 | IGLV4-69(458.9) | | IGLJ2(151) | 32524 | 51.57 | 183.24 |
|  | JeKo-1 | IGKV3-20(504.8) | | IGKJ2(149.7) | 40081 | 82.20 | 220.98 |
|  | Mino | IGLV3-19(403.4) | | IGLJ2(167) | 30091 | 89.73 | 128.06 |
|  | REC-1 | IGKV3-11(494.3) | | IGKJ1(148.3) | 107815 | 81.94 | 487.51 |
| Mycosis fungoides-Sezary syndrome | HuT 102 | TRAV12-2(368.4) | | TRAJ22(251) | 4967 | 53.71 | 30.17 |
|  | HuT 78 | TRBV13(405.5) | TRBD1(40) | TRBJ1-2(159) | 2023 | 55.39 | 12.52 |
|  | MJ | TRBV28(359.7) | TRBD1(30),  TRBD2(30) | TRBJ1-1(172.1) | 220 | 27.71 | 0.93 |
| Malignant histiocytosis | DEL | TRAV40(82.8) | | TRAJ4(48.8) | 409 | 50.49 | 2.08 |
| Peripheral T cell lymphoma unspecified | SUP-T11 | TRBV7-9(321.4) | TRBD1(25) | TRBJ1-1(207.2) | 6490 | 73.19 | 34.32 |
| Multiple myeloma | AMO-1 | IGKV3-20(359.8) | | IGKJ2(148.6) | 392010 | 84.00 | 2124.77 |
|  | EJM | IGLV1-40(466.7) | | IGLJ2(132.6) | 397799 | 74.06 | 2325.88 |
|  | HuNS1 | IGKV1-33(397.1) | | IGKJ4(141.8) | 27376 | 89.72 | 149.95 |
|  | JJN-3 | IGKV3-15(496.6) | | IGKJ4(137.6) | 123120 | 99.28 | 764.39 |
|  | KARPAS-620 | IGKV3-20(432) | | IGKJ4(132.8) | 403293 | 99.82 | 2438.67 |
|  | KE-97^(see the note at the end of the table)^ | IGLV3-21(362.3) | | IGLJ1(129.1) | 19374 | 87.92 | 118.79 |
|  | KHM-1B | IGLV1-44(400.4),  IGLV1-47(347.7) | | IGLJ3(128.1) | 505858 | 75.59 | 3555.13 |
|  | KMM-1 | IGLV1-51(238.2) | | IGLJ2(97.1),  IGLJ3(96.4) | 7983 | 91.92 | 55.69 |
|  | KMS-11 | IGKV3-15(462.2) | | IGKJ5(135.5) | 175273 | 89.33 | 1367.46 |
|  | KMS-12-BM | IGHV3-7(268.6) | IGHD4-23(40),  IGHD4-17(35) | IGHJ6(124.5) | 18169 | 96.80 | 107.94 |
|  | KMS-20 | IGKV3-15(419.4) | | IGKJ1(135) | 130874 | 73.21 | 579.98 |
|  | KMS-21BM | IGLV3-10(293.5) | | IGLJ3(110.3),  IGLJ2(106.1) | 126658 | 67.54 | 789.89 |
|  | KMS-26 | IGKV1-5(438) | | IGKJ2(124.9) | 396680 | 98.34 | 2120.45 |
|  | KMS-27 | IGKV2-40(412) | | IGKJ4(151.7) | 625058 | 61.50 | 4952.68 |
|  | KMS-28BM | IGLV3-10(276.5) | | IGLJ3(121.6),  IGLJ2(121.5) | 63869 | 98.96 | 404.77 |
|  | KMS-34 | IGKV3-15(420.3) | | IGKJ3(136.3) | 146773 | 99.56 | 832.90 |
|  | L-363 | IGLV2-23(373.9) | | IGLJ3(114) | 355114 | 99.81 | 2020.32 |
|  | LP-1 | IGLV3-21(397) | | IGLJ1(146.9) | 193100 | 86.94 | 1065.37 |
|  | MM1-S | IGLV2-14(347.7) | | IGLJ1(145.9) | 211289 | 99.59 | 1175.45 |
|  | MOLP-2 | IGLV2-11(251.1) | | IGLJ6(39.5) | 422942 | 96.49 | 2185.49 |
|  | MOLP-8 | IGLV2-23(237.3) | | IGLJ1(103.3) | 119627 | 58.73 | 534.68 |
|  | NCI-H929 | IGKV3-15(428.1) | | IGKJ1(149.2) | 117801 | 99.50 | 509.60 |
|  | OPM-2 | IGLV3-25(377.2),  IGLV3-16(306) | | IGLJ3(130.2) | 144947 | 99.66 | 911.41 |
|  | RPMI 8226 | IGLV2-14(300) | | IGLJ3(113.4) | 128512 | 99.58 | 872.88 |
|  | SK-MM-2 | IGKV1-39(307.2) | | IGKJ1(146) | 612942 | 99.90 | 3364.12 |
|  | U266B1 | IGLV2-8(317) | | IGLJ2(128.9),  IGLJ3(128.9) | 147989 | 87.81 | 1321.77 |

The relative clonal CPM value (count per million RNA sequencing reads) was calculated by dividing the clonal read counts by total RNA sequencing read counts numbers. The number inside the () indicates the score of the alignment.

#Note: U-937 is a well-recognized AML cell lines instead of a diffuse large B cell lymphoma, despite the fact that the cell line was original established from the pleural effusion of a patient with histiocytic lymphoma and it is still categorized as histiocytic lymphoma at ATCC (https://www.atcc.org/Products/All/CRL-1593.2.aspx) and CCLE database.

Note: KE-97 is a gastric cancer cell line. According to CCLE, it shares high similarly by SNP analysis with multiple myeloma KMS-18.

**Additional file 1: Table S2. The BCR/TCR repertoire of 164 blood cancer cell lines.**

|  | Cell line | Count | F % | All V hits | All D hits | All J hits | Seq. CDR3 | |
| --- | --- | --- | --- | --- | --- | --- | --- | --- |
| B-ALL | NALM-19 | 425 | 36.08 | IGHV1-3(252.4) | IGHD6-6(80) | IGHJ4(160.6) | CARDRV*QL_PPPLRDYW | |
|  |  | 42 | 3.57 | TRAV21(53.3) | | TRAJ27(61.3) | CAGWPHSTPGHLDIKSTF | |
|  |  | 39 | 3.31 | IGLV2-11(252.5) | | IGLJ6(47.4) | CCSISPTFSSFVF | |
|  | RCH-ACV | 10265 | 77.69 | IGHV1-3(218.6) | IGHD5-18(71), IGHD5-5(71) | IGHJ4(158), IGHJ5(151.2) | CASGPGRYSYGIDW | |
|  |  | 1752 | 13.26 | IGHV2-70(264.7) | IGHD3-3(130) | IGHJ4(158.9) | CALGFTIFGVVIIWGAYFDYW | |
|  |  | 379 | 2.87 | TRAV27(375.8) | | TRAJ49(214) | CAGADSGNQFYF | |
|  |  | 63 | 0.48 | TRAV8-7(67) | | TRAJ19(55) | CAGADRLQTGMRGAF | |
|  | MHH-CALL-3 | 1044 | 48.11 | IGHV3-15(248.6) | IGHD3-3(50) | IGHJ5(191.6) | CTTLGGGGFLDQSAAWFDPW | |
|  |  | 94 | 4.33 | IGLV3-1(401.3) | | IGLJ3(137.5) | CQAWDSSTVGKGVF | |
|  |  | 84 | 3.87 | IGHV3-15(284) | IGHD3-3(50) | IGHJ5(192.4) | CTTLGGGDFW_QSAAWFDPW | |
|  |  | 81 | 3.73 | IGLV1-44(377.5) | | IGLJ2(163.3) | CAAWDDSLNALVVF | |
|  |  | 50 | 2.30 | IGLV3-1(456.7) | | IGLJ2(157.6), IGLJ3(143.3) | CQAWDSSTGVF | |
|  |  | 43 | 1.98 | IGLV3-19(372.9) | | IGLJ2(161.8) | CNSRDSSGNHLGVVF | |
|  | MHH-CALL-4 | 214 | 14.33 | TRAV8-2(395.5) | | TRAJ8(223.6) | CVVGL_QKLVF | |
|  |  | 163 | 10.92 | IGHV2-26(318.4) | IGHD2-21(45), IGHD2-2(41), IGHD2-15(40) | IGHJ5(160) | CARIRPRAVR_CGSP*GPDPW | |
|  |  | 42 | 2.81 | TRAV2(430.4) | | TRAJ4(261.5) | CAVEAFSGGYNKLIF | |
|  | KASUMI-2 | 3491 | 60.82 | IGLV1-40(444.1) | | IGLJ3(155.9) | CQSYD_AGVF | |
|  |  | 1716 | 29.90 | IGHV3-7(298.3) | IGHD3-10(100) | IGHJ4(142), IGHJ5(134.1) | CARDPRRVLWFGELTQIIGRDW | |
|  | MUTZ-5 | 1632 | 76.30 | IGLV2-34(350.5) | | IGLJ2(163.4) | CSSYA_HLVVF | |
|  | SUP-B15 | 2739 | 71.74 | IGHV3-53(321.7) | IGHD2-8(90) | IGHJ6(157.3) | CTRVARGWCMLYRYYYYGMDVW | |
|  |  | 50 | 1.31 | IGKV3-20(302.9) | | IGKJ4(115.4) | CQQYAGSALTF | |
|  |  | 47 | 1.23 | TRAV8-7(68) | | TRAJ19(54.7) | CAGADRLQTGMRGAF | |
|  |  | 42 | 1.10 | IGHV3-32(46.1) | | IGHJ6(220.8) | YTGTTT_GMDVW | |
|  |  | 40 | 1.05 | IGKV2-30(545.3) | | IGKJ5(156.5) | CMQE_RITF | |
|  |  | 32 | 0.84 | IGLV3-1(364.5) | | IGLJ1(148.1) | CQAWDSSSWVF | |
|  | 697 | 1373 | 65.38 | IGHV2-26(301.6) | IGHD2-2(120) | IGHJ4(157.8) | CARIRPYCSSTSCYNESTAFDYW | |
|  |  | 88 | 4.19 | IGHV4-34(205.2), IGHV4-4(192.4) | IGHD3-22(115) | IGHJ2(175.9) | CAREHPLVRFG*MLL***WLLVNWYFDLW | |
|  |  | 46 | 2.19 | IGLV11-55(88.2), IGLV5-48(75) | | IGLJ7(40) | CAMG_PQF | |
|  | SEM | 32 | 6.53 | TRAV8-7(68.6) | | TRAJ19(54.7) | CAGADRLQTGMRGAF | |
|  | Reh | 100 | 16.21 | IGHV3-15(347.8) | IGHD3-10(70) | IGHJ6(185.4) | CTTGMVRGVI_YYYYGMDVW | |
|  | KOPN-8 | 6012 | 75.41 | IGHV6-1(364.4) | IGHD1-1(46) | IGHJ4(195.6) | CARGWDNWNDFDYW | |
|  |  | 1015 | 12.73 | IGLV4-3(496.9) | | IGLJ3(136.2) | CGESHTIDGQVGWVF | |
|  |  | 488 | 6.12 | IGHV1-2(318) | IGHD2-2(143) | IGHJ5(116), IGHJ4(103.5) | CARGV*EDIVVVPAAMQAYW | |
|  |  | 33 | 0.41 | TRAV8-7(67) | | TRAJ19(55.2) | CAGADRLQTGMRGAF | |
|  | RS4;11 | 361 | 26.05 | IGHV6-1(340.8) | IGHD1-20(45), IGHD1-7(45) | IGHJ4(184.4) | CAREP*LELFDYW | |
|  |  | 241 | 17.39 | IGHV3-20(252.2) | IGHD2-8(110) | IGHJ5(189.6) | CARD*SRY*W_VCYTDWFDPW | |
|  |  | 173 | 12.48 | IGLV11-55(92.2) | | IGLJ7(39.8) | CAMG_PQF | |
|  |  | 73 | 5.27 | TRDV2(365) | TRDD3(61) | TRDJ2(61) | CACDGRGLGDTDSGNTPLVF | |
|  |  | 37 | 2.67 | TRAV8-7(67) | | TRAJ19(53.4) | CAGADRLQTGMRGAF | |
|  |  | 36 | 2.60 | IGLV4-3(534.6) | | IGLJ3(148) | CGESHTIDG_SRLRFWVF | |
|  | NALM-6 | 15670 | 74.70 | IGLV2-14(382.1) | | IGLJ7(134.6) | CSSYTSS_ALGAVF | |
|  |  | 2479 | 11.82 | IGLV4-3(452.5) | | IGLJ3(118.7) | CGESHTIDGQ_RLQAPGGVF | |
|  |  | 2229 | 10.63 | IGHV1-69(290.4) | IGHD3-10(30), IGHD2-21(26), IGHD2-8(26) | IGHJ6(211.6) | CARDRRGEWPPSDYYYYYMDVW | |
|  | BDCM | 32303 | 69.88 | IGLV4-60(399.1) | | IGLJ2(141.6), IGLJ3(117.6) | CETWDIHGVF | |
|  |  | 13228 | 28.62 | IGHV3-23(307.7) | IGHD1-7(35), IGHD2-8(35), IGHD3-16(35) | IGHJ4(174.2) | CAKYLTERYRVDYW | |
|  |  | 75 | 0.16 | IGHVIII-67-3(133.5) | IGHD6-13(95) | IGHJ4(157.3), IGHJ5(142.5) | ITVREPAPDH_DYSSSWYAYW | |
|  |  | 40 | 0.09 | TRAV8-7(67) | | TRAJ19(55) | CAGADRLQTGMRGAF | |
| T-ALL | MOLT-16 | 15457 | 50.22 | TRBV20-1(349.6) | TRBD1(35) | TRBJ2-3(200.9) | CSAGESGGVTDTQYF | |
|  |  | 5990 | 19.46 | TRAV12-1(384.6) | | TRAJ9(225.9) | CVVNGTGGFKTIF | |
|  |  | 3762 | 12.22 | TRAV3(320.5) | | TRAJ5(270.1) | CAVRDPDTGRRALTF | |
|  |  | 2117 | 6.88 | TRGV8(347.9) | | TRGJ1(192.9), TRGJ2(192.9) | CATWTDRIYYKKLF | |
|  |  | 2011 | 6.53 | TRGV8(423.4) | | TRGJP2(197.1) | CAPLE_WIKTF | |
|  |  | 837 | 2.72 | TRBV27(319.3) | TRBD1(48) | TRBJ1-1(184.9) | CASTDPDR_EWTEAFF | |
|  | JURKAT | 10591 | 86.02 | TRBV12-3(429.5), TRBV12-4(403.8) | TRBD1(25) | TRBJ1-2(203.4) | CASSFSTCSANYGYTF | |
|  |  | 1187 | 9.64 | TRAV8-4(346.9) | | TRAJ3(208.2) | CAVSDLEPNSSASKIIF | |
|  | TALL-1 | 9397 | 82.88 | TRBV20-1(399.8) | | TRBJ2-5(186.1) | CSDPSRETQYF | |
|  |  | 1087 | 9.59 | TRBV5-6(368.8) | TRBD2(60) | TRBJ2-3(181.6) | CASSGD*RVRDTQYF | |
|  |  | 280 | 2.47 | TRAV8-4(395.9) | | TRAJ4(262.2) | CAVSAISGGYNKLIF | |
|  |  | 67 | 0.59 | IGKV3-20(324.9) | | IGKJ4(123.8) | CQQYAGSALTF | |
|  |  | 66 | 0.58 | TRGV5(338.6) | | TRGJ1(246.7), TRGJ2(246.7) | CATWSNYYKKLF | |
|  |  | 39 | 0.34 | TRAV8-7(67) | | TRAJ19(55) | CAGADRLQTGMRGAF | |
|  | MOLT-3 | 38063 | 90.81 | TRBV20-1(404.8) | | TRBJ2-1(183) | CSARESTSDPKNEQFF | |
|  |  | 2913 | 6.95 | TRBV10-3(445.2) | | TRBJ2-5(182.6) | CAISEPTG_SEETQYF | |
|  |  | 231 | 0.55 | TRAV1-1(309.2) | | TRAJ33(267.3) | CAVRDHPW_SNYQLIW | |
|  |  | 97 | 0.23 | TRAV1-1(288.2) | | TRAJ24(233.6) | CAVKMEQ_WGKLQF | |
|  | PEER | 21624 | 89.06 | TRBV4-2(326.7) | | TRBJ2-3(186.9) | CASSQETLPLRLGTQYF | |
|  |  | 1165 | 4.80 | TRDV1(443.6) | TRDD3(25) | TRDJ1(208.4) | CALGTGVRGLQDTDKLIF | |
|  |  | 653 | 2.69 | TRBV21-1(365.5) | | TRBJ2-7(186) | CASTVEQ_TPYEQYF | |
|  |  | 193 | 0.79 | TRGV8(407.1) | | TRGJ1(205.8), TRGJ2(205.8) | CATWKDYYKKLF | |
|  |  | 74 | 0.30 | TRGV9(449) | | TRGJ1(162.4), TRGJ2(162.4) | CAFRP_KKLF | |
|  |  | 32 | 0.13 | TRAV8-7(67) | | TRAJ19(55) | CAGADRLQTGMRGAF | |
|  | MOLT-13 | 1345 | 58.20 | TRBV10-1(322.3) | TRBD2(28), TRBD1(25) | TRBJ1-1(203.1) | CASRRVRRDRNTEAFF | |
|  |  | 365 | 15.79 | TRDV1(437.7) | | TRDJ1(228.1) | CALGEPGGYTDKLIF | |
|  |  | 134 | 5.80 | TRGV3(395.5), TRGV5(320) | | TRGJ1(178.6), TRGJ2(178.6) | CATWDRPRLKKLF | |
|  |  | 32 | 1.38 | TRGV8(429) | | TRGJP1(238.2) | CATWDP_GWFKIF | |
|  | KE-37 | 656 | 56.21 | TRBV3-2(375.5) | TRBD1(60) | TRBJ1-5(199.5) | CASSQDSGTG_RVGNQPQHF | |
|  | ALL-SIL | 2364 | 51.42 | TRGV7(395.9) | | TRGJ1(192.1), TRGJ2(192.1) | CATWGSG_YYKKLF | |
|  |  | 909 | 19.77 | TRBV18(390) | TRBD1(26) | TRBJ2-1(171.9) | CASSPMEK_GHKGEQFF | |
|  |  | 482 | 10.49 | TRBV7-9(386.4) | TRBD1(30) | TRBJ2-7(173.4) | CASSLDT_WYEQYF | |
|  |  | 221 | 4.81 | TRGV9(420) | | TRGJ1(211), TRGJ2(211) | CALWR*_YYKKLF | |
|  |  | 86 | 1.87 | TRDV2(419.1) | | TRDJ1(204.8) | CACDK_DKLIF | |
|  | HPB-ALL | 23926 | 47.87 | TRBV5-5(379.4) | | TRBJ2-5(172.3) | CASSSRKTQYF | |
|  |  | 9451 | 18.91 | TRAV19(418.9) | | TRAJ3(251.8) | CALDSSASKIIF | |
|  |  | 7628 | 15.26 | TRAV5(371.8) | | TRAJ3(258.8) | CAEYSSASKIIF | |
|  |  | 6773 | 13.55 | TRBV7-3(355.5) | TRBD2(30) | TRBJ2-5(183) | CASSPR_RETQYF | |
|  |  | 716 | 1.43 | TRGV3(376.9) | | TRGJ1(207), TRGJ2(207) | CATWDRQ_NYYKKLF | |
|  |  | 637 | 1.27 | TRGV3(314.4) | | TRGJ1(175.7), TRGJ2(175.7) | CATWDKRCL_LTGVYKKLF | |
|  |  | 61 | 0.12 | TRAV35(66) | | TRAJ15(24.5) | CMIVGSPELTF | |
|  |  | 35 | 0.07 | IGKV4-1(519.9) | | IGKJ3(159.3) | CQQYYSTPQFTF | |
|  | P12-ICHIKAWA | 14632 | 77.19 | TRBV6-5(392.3) | | TRBJ2-1(198.2) | CASSIRTPHPHYNEQFF | |
|  |  | 3297 | 17.39 | TRBV10-1(351.8) | | TRBJ2-3(218.5) | CASRESG_TDTQYF | |
|  |  | 276 | 1.46 | TRGV9(457) | | TRGJ1(157.6), TRGJ2(157.6) | CALWEVSA*KLF | |
|  |  | 70 | 0.37 | TRDV1(443.7) | TRDD3(30) | TRDJ1(198.1) | CALGELRPL_GRSTDKLIF | |
|  |  | 39 | 0.21 | TRBV21-1(34.2), TRBV3-1(30.3), TRBV3-2(30.3) | TRBD2(27), TRBD1(26) | TRBJ2-4(213.9) | CAASRGC_AKNIQYF | |
|  | PF-382 | 16814 | 90.26 | TRBV20-1(391.2) | TRBD2(65) | TRBJ2-1(178.3) | CSATSGLAGLNEQFF | |
|  |  | 806 | 4.33 | TRBV7-5(342.9) | TRBD1(25), TRBD2(25) | TRBJ2-7(169.6) | CISVPEAQR_FRASEQYF | |
|  |  | 478 | 2.57 | TRAV8-4(368) | | TRAJ3(242.6) | CAVSEEQ_ASKIIF | |
|  |  | 40 | 0.21 | TRBV7-1(108.9) | TRBD1(25), TRBD2(25) | TRBJ2-7(203.5) | CARSSEAFRASEQYF | |
|  |  | 39 | 0.21 | TRGV10(480.4) | | TRGJ1(183.2), TRGJ2(183.2) | CAAWDEGYKKLF | |
|  | Loucy | 12952 | 60.90 | TRBV5-6(334.5) | TRBD1(35) | TRBJ2-1(209.9) | CASSRGEPPWTYNEQFF | |
|  |  | 6785 | 31.91 | TRAV23DV6(397.1) | | TRDJ2(236.7) | CAASKAAGLTAQLFF | |
|  |  | 572 | 2.69 | TRGV9(388.4) | | TRGJ1(228), TRGJ2(228) | CALWEPPPSNYYKKLF | |
|  |  | 350 | 1.65 | TRBV20-1(398.8) | TRBD1(25) | TRBJ2-2(217.9) | CSARAEAQ_NTGELFF | |
|  |  | 96 | 0.45 | TRGV2(370.5) | | TRGJ1(137.1), TRGJ2(137.1) | CATWGSQDFTF | |
|  | DND-41 | 4868 | 84.46 | TRBV18(439.2) | TRBD1(50) | TRBJ1-2(172.7) | CASSPGTGRYGYTF | |
|  |  | 271 | 4.70 | TRBV6-3(256.2), TRBV6-2(255.8) | TRBD1(35), TRBD2(30) | TRBJ2-7(205.6) | CASSYPRQG_ERGSYEQYF | |
|  |  | 183 | 3.17 | TRDV1(333.2) | TRDD2(36) | TRDJ1(219) | CALGEAFRPSRTFTDKLIF | |
|  |  | 72 | 1.25 | IGKV3-20(330.3) | | IGKJ4(125.5) | CQQYAGSALTF | |
|  | SUP-T1 | 12869 | 93.19 | TRBV9(416.8) | | TRBJ2-1(152.4) | CASSVGGSLKQFF | |
|  |  | 130 | 0.94 | TRGV3(369.3) | | TRGJ1(232.3), TRGJ2(232.3) | CATWRTNYYKKLF | |
|  |  | 71 | 0.51 | TRAV1-1(471.7) | | TRAJ12(216.7) | CAVPP*W_SSYKLIF | |
|  |  | 37 | 0.27 | IGKV3-20(292.6) | | IGKJ4(120) | CQQYAGSALTF | |
|  | RPMI-8402 | 15614 | 93.11 | TRBV15(266.9) | TRBD1(26), TRBD2(25) | TRBJ1-5(230.1) | CATSRDGKGSNQPQHF | |
|  |  | 375 | 2.24 | TRBV19(336.2) | TRBD1(32), TRBD2(28) | TRBJ2-7(198) | CAY*GRV_LSYEQYF | |
|  |  | 153 | 0.91 | TRGV10(445.9) | | TRGJ1(204.1), TRGJ2(204.1) | CAAWGI_YKKLF | |
|  |  | 132 | 0.79 | TRGV4(415.6) | | TRGJ1(209.2), TRGJ2(209.2) | CATWDGASTNYYKKLF | |
| AML | PL-21 | 44 | 7.80 | TRAV8-7(67) | | TRAJ19(55) | CAGADRLQTGMRGAF | |
|  | ME-1 | 54 | 12.53 | TRAV8-7(67.9) | | TRAJ19(55) | CAGADRLQTGMRGAF | |
|  | TF-1 | 70 | 9.42 | TRAV8-7(69.1) | | TRAJ19(54.9) | CAGADRLQTGMRGAF | |
|  | Kasumi-6 | 59 | 11.07 | IGKV2-40(387.3) | | IGKJ4(148.6) | SMQLF |  |
|  | OCI-M1 | 37 | 7.69 | TRAV8-7(65.2) | | TRAJ19(55) | CAGADRLQTGMRGAF | |
|  | AML-193 | 71 | 13.79 | IGKV1-5(370.7) | | IGKJ2(124.4) | CQQYKTYTF | |
|  |  | 40 | 7.77 | IGLV1-40(361.6) | | IGLJ2(132), IGLJ3(106) | CQSYDGSLSAVVF | |
|  |  | 35 | 6.80 | IGKV3-15(264.1) | | IGKJ3(157.1) | CQQYKTWLPLTF | |
|  | P31/FUJ | 145 | 20.60 | TRGV10(416.6) | | TRGJP2(259.7) | CAAWAYI_SDWIKTF | |
|  | F-36P | 46 | 7.28 | IGLV3-21(382.1) | | IGLJ1(144.7) | CQVWDSTSDHYVF | |
|  |  | 39 | 6.17 | TRAV8-7(67) | | TRAJ19(55.1) | CAGADRLQTGMRGAF | |
|  |  | 32 | 5.06 | IGKV1-5(407.2) | | IGKJ2(108.1) | CQQYKTYTF | |
|  | NB-4 | 42 | 7.58 | IGKV1-5(417.7) | | IGKJ2(120.5) | CQQYKTYTF | |
|  | SKM-1 | 34 | 6.80 | TRAV8-7(68.5) | | TRAJ19(55) | CAGADRLQTGMRGAF | |
|  | KASUMI-1 | 63 | 10.08 | TRAV8-7(69) | | TRAJ19(54.7) | CAGADRLQTGMRGAF | |
|  |  | 54 | 8.64 | IGKV3-20(284.9) | | IGKJ4(99.7) | CQQYAGSALTF | |
| Adult T cell lymphoma-leukaemia | HH | 607 | 40.25 | TRBV2(301.6) | TRBD2(25) | TRBJ2-3(213.2) | CASKTTQSTDTQYF | |
|  |  | 280 | 18.57 | TRAV13-2(385.3) | | TRAJ36(244.5) | CAENIGTGANNLFF | |
|  |  | 76 | 5.04 | TRBV19(325.2) | | TRBJ2-2(216.5) | CAPSN*HTGELFF | |
| Anaplastic large cell lymphoma | SU-DHL-1 | 185 | 18.39 | TRAV40(82) | | TRAJ4(50.5) | CLLGSISLGILSQ | |
|  |  | 131 | 13.02 | TRBV27(333.4) | TRBD1(25) | TRBJ2-3(197.3) | CASSFSRGTDTQYF | |
|  |  | 56 | 5.57 | TRAV8-7(68.7) | | TRAJ19(54.7) | CAGADRLQTGMRGAF | |
|  | Ki-JK | 210 | 29.66 | TRAV40(82.4) | | TRAJ4(50.9) | CLLGSISLGILSQ | |
|  | KARPAS-299 | 723 | 55.02 | TRBV20-1(397.8) | TRBD2(31) | TRBJ2-7(170) | CSARAQIGSSPLEQYF | |
|  |  | 113 | 8.60 | TRAV40(82.4) | | TRAJ4(54.3) | CLLGSISLGILSQ | |
|  | SR-786 | 176 | 15.20 | TRAV40(82.5) | | TRAJ4(49.8) | CLLGSISLGILSQ | |
|  |  | 87 | 7.51 | IGKV3-20(314.6) | | IGKJ4(118.1) | CQQYAGSALTF | |
|  |  | 56 | 4.84 | TRAV8-2(59) | | TRAJ26(67.7) | CVVGITMIDLYPRDSWNFVF | |
|  |  | 41 | 3.54 | TRAV28(71) | | TRAJ58(54.7) | CWGVPPAQPPHVPPCASSGF | |
|  |  | 38 | 3.28 | TRAV8-7(68.3) | | TRAJ19(55) | CAGADRLQTGMRGAF | |
|  | SUP-M2 | 224 | 21.75 | TRAV40(82) | | TRAJ4(50.6) | CLLGSISLGILSQ | |
|  |  | 169 | 16.41 | TRAV8-4(331.5) | | TRAJ11(220.9) | CAVSDRGYSTLTF | |
|  |  | 68 | 6.60 | TRAV14DV4(284.6) | | TRAJ39(243.8) | CAMREGQ_AGNMLTF | |
| B cell lymphoma unspecified | RI-1 | 104681 | 81.36 | IGKV4-1(428.1) | | IGKJ2(148.8) | CQQYYSVPYTF | |
|  |  | 23413 | 18.20 | IGHV3-7(241.6) | IGHD3-10(40), IGHD3-16(36) | IGHJ4(176.2) | CARKCTPERGKLGFFDYW | |
|  | MC116 | 29091 | 66.03 | IGLV2-14(408.8) | | IGLJ2(146.5) | CSSYTSSSTLVVF | |
|  |  | 14175 | 32.18 | IGHV1-2(315.2) | IGHD1-26(60) | IGHJ4(161.7) | CARGDSGSYHYW | |
|  |  | 284 | 0.64 | IGKV5-2(451.7) | | IGKJ3(162.2) | CLQHDNFPLFTF | |
|  | RL | 12391 | 72.33 | IGLV2-11(228.2) | | IGLJ1(118.5) | CCSYGVNNSFPYVF | |
|  |  | 4225 | 24.66 | IGHV3-23(192.9) | IGHD2-15(40), IGHD2-21(40), IGHD3-22(35) | IGHJ4(168.1) | CAKHSGDYFFDSW | |
|  | NU-DUL-1 | 10266 | 40.59 | IGLV1-40(224.9) | | IGLJ2(100.5) | CQSSDDRFIIF | |
|  |  | 3738 | 14.78 | IGLV1-44(426.9), IGLV1-47(346.4) | | IGLJ3(154.2) | CAAWDDSLNGWVF | |
|  |  | 3005 | 11.88 | IGLV4-60(424.5) | | IGLJ3(151.5) | CETWDSNTGVF | |
|  |  | 2974 | 11.76 | IGLVI-70(464.4) | | IGLJ3(154) | CSTWDYS_SVFWVF | |
|  |  | 2560 | 10.12 | IGHV4-59(178.1), IGHV4-61(170.6) | IGHD7-27(35), IGHD2-21(34), IGHD3-9(30) | IGHJ5(126.2) | CARGGGTGVDNWFGLW | |
|  |  | 1180 | 4.67 | IGLV1-44(444.2), IGLV1-47(372.5) | | IGLJ3(136.5), IGLJ2(110.7) | CAAWDDSLNGPVF | |
|  |  | 507 | 2.00 | IGLV9-49(492.2) | | IGLJ3(136.4) | CGADHGSGSNFVWVF | |
|  |  | 154 | 0.61 | IGLV6-57(451.3) | | IGLJ3(149.6) | CQSYDSSNHWVF | |
|  |  | 71 | 0.28 | IGLV6-57(434) | | IGLJ3(151.8) | CQSYDSSNWVF | |
|  |  | 56 | 0.22 | IGLV1-51(386.6) | | IGLJ3(142.4), IGLJ2(119.2) | CGTWDSSLSVF | |
|  |  | 42 | 0.17 | IGLV1-51(418.7) | | IGLJ3(162.9) | CGTWDSSLSAHWVF | |
|  | BCP-1 | 36 | 7.76 | IGHV3-23(303.7) | IGHD3-10(56) | IGHJ4(187.5) | CAKDELVARGVFDYW | |
|  | JM1 | 10946 | 48.32 | IGLV3-1(426) | | IGLJ6(155.9) | CQAWD_QPNVF | |
|  |  | 7877 | 34.78 | IGLV3-10(464) | | IGLJ6(134.6) | CYSTDSS_VIIANVF | |
|  |  | 1135 | 5.01 | IGLV8-61(59.9) | | IGLJ7(137.5) | CV_VF |  |
|  |  | 90 | 0.40 | TRAV8-7(68.1) | | TRAJ19(54.8) | CAGADRLQTGMRGAF | |
|  |  | 67 | 0.30 | IGKV1-5(531.3) | | IGKJ1(148.5) | CQQYNSYWTF | |
|  |  | 57 | 0.25 | IGKV1-5(478.5) | | IGKJ1(157.2) | CQQYNSYSWTF | |
|  |  | 36 | 0.16 | IGKV5-2(518) | | IGKJ2(152.3) | CLQHDNFPYTF | |
|  |  | 33 | 0.15 | IGKV3-20(305.5) | | IGKJ4(109.5) | CQQYAGSALTF | |
|  |  | 32 | 0.14 | IGLV4-60(75) | | IGLJ1(45) | CETWGVNG_DGYFGGHE | |
|  | HT | 18074 | 89.54 | IGKV3-11(408.4) | | IGKJ5(155.6) | CQQRTNWPITF | |
|  |  | 1488 | 7.37 | IGHV3-53(202.9) | IGHD1-1(30), IGHD7-27(30) | IGHJ4(158.1) | CARASFAT_*LYFDSW | |
| Blast phase CML | CML-T1 | 3251 | 62.36 | TRBV19(336.7) | TRBD2(61) | TRBJ2-5(188.4) | CASTTRTSGGQETQYF | |
|  |  | 746 | 14.31 | TRAV1-2(358) | | TRAJ3(269.9) | CAVRPGYSSASKIIF | |
|  |  | 592 | 11.36 | TRAV2(369.3) | | TRAJ3(247.7) | CAVEEHHPPYSSASKIIF | |
|  |  | 100 | 1.92 | TRGV4(478.9) | | TRGJ1(203.1), TRGJ2(203.1) | CATWDGTR_DYYKKLF | |
|  | KCL-22 | 158 | 22.25 | IGKV2-40(397.6) | | IGKJ4(150.1) | SMQLF |  |
|  |  | 96 | 13.52 | IGKV1-39(420.3) | | IGKJ1(169.3) | CQQSYSSPQWTF | |
|  | KYO-1 | 71 | 14.49 | TRAV8-7(67.7) | | TRAJ19(55) | CAGADRLQTGMRGAF | |
|  | MOLM-6 | 41 | 7.52 | TRAV8-7(67) | | TRAJ19(55) | CAGADRLQTGMRGAF | |
|  | BV-173 | 764 | 57.83 | IGHV3-21(289.7) | IGHD2-15(105) | IGHJ3(181.4) | CASQIL*WW*_PYRGAFDIW | |
|  |  | 157 | 11.88 | IGKV2-29(427.9) | | IGKJ3(166.7) | *MQGIH_SSLFTF | |
|  |  | 35 | 2.65 | TRAV8-7(69) | | TRAJ19(54.9) | CAGADRLQTGMRGAF | |
|  | NALM-1 | 5346 | 88.35 | IGHV3-9(297.6) | IGHD2-21(95) | IGHJ6(146) | CIVVVTATEGGMDVW | |
|  |  | 57 | 0.94 | IGLV5-48(87.8) | | IGLJ7(40.2) | CAMG_PQF | |
|  |  | 48 | 0.79 | IGHV4-31(396) | | IGHJ5(170.6) | CARDGDPW | |
|  | K-562 | 46 | 7.44 | TRAV8-7(71.9) | | TRAJ19(54.9) | CAGADRLQTGMRGAF | |
| Burkitt lymphoma | Raji | 38181 | 85.01 | IGKV3-20(374) | | IGKJ2(138.3) | CQQYGSSTLFTF | |
|  |  | 5925 | 13.19 | IGHV3-21(172.2), IGHV3-7(156.7) | IGHD3-22(65) | IGHJ4(138.7) | CARQRNDFSDNNSYYSNFDFW | |
|  |  | 74 | 0.16 | IGHV3-21(221.5), IGHV3-7(178.3) | IGHD3-22(79) | IGHJ4(135.7) | CARQSNDFSDNNGYYSNFDFW | |
|  |  | 34 | 0.08 | IGHV3-7(114.8), IGHV3-21(98.5) | IGHD3-22(65) | IGHJ4(133.3) | CARQRNDFSDNNSYYSNFDFWGHFDFW | |
|  | Daudi | 30696 | 70.29 | IGKV1-39(333) | | IGKJ4(130) | CQQNYNFSFTF | |
|  |  | 12332 | 28.24 | IGHV3-74(162.7) | IGHD3-3(35), IGHD3-9(35), IGHD2-8(30) | IGHJ4(153.7) | CVRGNGQKCFDYW | |
|  |  | 97 | 0.22 | IGHV3-25(49) | IGHD3-3(35), IGHD3-9(35), IGHD2-8(30) | IGHJ4(171.1) | CKRKWS_KCFDYW | |
|  | BL-70 | 21725 | 69.76 | IGKV1-39(475.4) | | IGKJ4(172.8) | CQQSYSTPLTF | |
|  |  | 8828 | 28.35 | IGHV3-53(256.6) | IGHD3-22(55), IGHD3-3(55) | IGHJ2(188.7) | CARTPDISGYYYRYFDLW | |
|  |  | 79 | 0.25 | IGHV3-62(37) | | IGHJ2(181.3) | WYFDLW |  |
|  |  | 58 | 0.19 | IGHV3-33-2(53.3), IGHV3-79(50.1) | IGHD6-19(31), IGHD6-25(31) | IGHJ2(201.4) | CMGEAGGQRLYYWYFDLW | |
|  | NAMALWA | 67629 | 85.38 | IGLV4-60(453.4) | | IGLJ3(140.4) | CETWDSNTRVF | |
|  |  | 4365 | 5.51 | IGHV4-28(250.5), IGHV4-61(235.2), IGHV4-4(222.3) | IGHD3-3(100) | IGHJ4(148.5) | CARRNYDFWSGGDGPFDYW | |
|  |  | 1471 | 1.86 | IGLV8-61(434.2) | | IGLJ3(145.2), IGLJ2(128.3) | CVLYMGSGILVF | |
|  |  | 1348 | 1.70 | IGLV6-57(447.3) | | IGLJ3(146) | CQSYDSSNHWVF | |
|  |  | 489 | 0.62 | IGHV1-69(162.7), IGHV1-8(144.8) | IGHD5-24(57), IGHD4-11(51), IGHD4-4(51) | IGHJ2(201.2) | CAGPGKGRHDY_IKDYWYFDLW | |
|  |  | 437 | 0.55 | IGLV2-8(407.4) | | IGLJ3(143.8) | CSSYAGSNNLVF | |
|  |  | 208 | 0.26 | IGLV4-60(442.8) | | IGLJ3(141.5), IGLJ2(136.3) | CETWDSNTRVF | |
|  |  | 120 | 0.15 | IGLV4-60(479.5) | | IGLJ3(135.2) | *DLGQ_NTRVF | |
|  |  | 116 | 0.15 | IGLV4-60(431.9) | | IGLJ3(136.6) | CETWD_NTRVF | |
|  |  | 101 | 0.13 | IGLV3-1(485.7) | | IGLJ3(124.6) | CQAWD_AALVF | |
|  |  | 99 | 0.12 | IGLV3-1(452.3) | | IGLJ3(144.6) | CQAWDSSTWVF | |
|  |  | 97 | 0.12 | IGKV4-1(521.6) | | IGKJ4(142.4) | CQQYY_VLLTF | |
|  |  | 89 | 0.11 | IGLV3-1(499) | | IGLJ3(138.8) | CQAWDSSTAVF | |
|  |  | 87 | 0.11 | IGLV2-8(380.4) | | IGLJ3(154.3) | CSSYAGSNNWVF | |
|  |  | 69 | 0.09 | IGLV3-1(435.3) | | IGLJ3(147.2), IGLJ2(118.2) | CQAWDSSTAWVF | |
|  |  | 57 | 0.07 | IGHV1-69(168.1), IGHV1-8(157.3) | IGHD5-24(57), IGHD4-11(51), IGHD4-4(51) | IGHJ2(177.4) | CAGPGKGRHDY_IKDYWYFDLW | |
|  |  | 55 | 0.07 | IGLV3-1(499.8) | | IGLJ3(127.2) | CQAWDSSTVF | |
|  |  | 52 | 0.07 | IGLV6-57(438.2) | | IGLJ3(148.6) | CQSYDNSNHWVF | |
|  |  | 52 | 0.07 | IGLV3-1(466.8) | | IGLJ3(128.7) | CQAWD_QHWVF | |
|  |  | 46 | 0.06 | IGLV5-45(438.5) | | IGLJ3(147.6) | CMIWHSSAWVF | |
|  |  | 44 | 0.06 | IGLV3-1(460.1) | | IGLJ2(151.9) | CQAWDSSTVVF | |
|  |  | 41 | 0.05 | IGLV4-3(535.8) | | IGLJ3(132.2) | CGESHTIDGQVGWVF | |
|  |  | 35 | 0.04 | IGLV2-8(401.1) | | IGLJ3(156.4) | CSSYAG_ATIWVF | |
|  |  | 32 | 0.04 | IGLV3-19(432.8) | | IGLJ3(144.6) | CNSRDSSGNHWVF | |
|  | GA-10 | 28987 | 87.40 | IGKV2-28(429.9) | | IGKJ2(173.6) | CMQALQTPPMYTF | |
|  |  | 1371 | 4.13 | IGHV4-34(229.9) | IGHD2-21(31), IGHD3-22(31) | IGHJ6(164.5) | CAKGSIEVLAGAYYYYGLDVW | |
|  |  | 1144 | 3.45 | IGKV5-2(337.2) | | IGKJ4(175.5) | CLQHDN_PSQLTF | |
|  |  | 833 | 2.51 | IGHV4-34(249.5) | IGHD3-16(35), IGHD3-9(33), IGHD2-21(31) | IGHJ6(159.5) | CAKGSITVLAGAYYYYGLDVW | |
|  |  | 199 | 0.60 | IGKV5-2(339.9) | | IGKJ4(153.6) | CLQHDNFPI*GCYILLLSTT**FPSQLTF | |
|  | BL-41 | 34963 | 56.47 | IGKV3-20(413.8) | | IGKJ2(159) | CQQYSTSPYTF | |
|  |  | 17359 | 28.04 | IGKV4-1(516.7) | | IGKJ5(149.9) | CQQYYSTPITF | |
|  |  | 9068 | 14.65 | IGHV3-30(278.4), IGHV3-33(233.5), IGHV3-66(224.3) | IGHD5-24(30) | IGHJ4(187.8) | CAKARHPETYYFFDYW | |
|  |  | 71 | 0.11 | IGLV3-1(483.1) | | IGLJ2(138.2) | CQAWDSSTVVF | |
|  | ST486 | 18336 | 85.15 | IGKV2-28(478.2) | | IGKJ2(124.4) | CMQALQTPRTF | |
|  |  | 2213 | 10.28 | IGHV3-30(177.7) | IGHD3-3(92) | IGHJ4(136.5), IGHJ5(117.5) | CAKGEEKTGHSGFWSGYLW | |
|  |  | 171 | 0.79 | IGKV3-20(300.3) | | IGKJ4(117.2) | CQQYAGSALTF | |
|  |  | 52 | 0.24 | IGKV3-31(50) | | IGKJ5(186) | CG_TF |  |
|  |  | 40 | 0.19 | TRAV8-7(67) | | TRAJ19(55) | CAGADRLQTGMRGAF | |
|  |  | 36 | 0.17 | TRBV1(79), TRBV4-1(74.6), TRBV4-3(74.6) | TRBD1(50), TRBD2(45) | TRBJ1-3(60.8) | CPSTNKLGCCVNV_GRQHWSGTPVWPP | |
|  |  | 31 | 0.14 | IGLV2-8(271.7) | | IGLJ2(127.4), IGLJ3(127.4) | CSSYAGSNSLIF | |
|  | EB1 | 11557 | 57.29 | IGLV1-51(309.4) | | IGLJ2(141.7) | CGTWDNRLSVVIF | |
|  |  | 5962 | 29.55 | IGLV1-51(348.2) | | IGLJ2(147.6) | CGTWDNSLSVVIF | |
|  |  | 671 | 3.33 | IGHV4-34(182.9) | IGHD2-15(67) | IGHJ5(154.2) | CARNVGYCIGSKCLIKWFAPW | |
|  |  | 551 | 2.73 | IGKV1-39(405.1) | | IGKJ4(133.4) | CQCGY_VHLTF | |
|  |  | 281 | 1.39 | IGHV4-34(199.6) | IGHD2-15(67), IGHD2-8(60) | IGHJ5(161.3) | CARGVGYCTGSKCLIKWFAPW | |
|  |  | 157 | 0.78 | IGLV1-51(338) | | IGLJ2(137.4), IGLJ3(117.6) | CGTGDNSLSVVIF | |
|  |  | 108 | 0.54 | IGHV4-34(115.9) | IGHD2-15(67), IGHD2-8(60) | IGHJ5(157.8) | CARNVGYCTGSKCLIKWFAPW | |
|  |  | 51 | 0.25 | IGHV4-34(160.6) | IGHD2-15(67), IGHD2-8(60) | IGHJ5(161.5) | CARGVGYCTGSKCLIKCFAPW | |
|  |  | 48 | 0.24 | IGHV4-34(188.7) | IGHD2-15(67), IGHD2-8(60) | IGHJ5(154) | SARGVGYCTGSKCLIKWFAPW | |
|  |  | 38 | 0.19 | IGLV1-51(283.9) | | IGLJ2(138.1) | CGTWDNRLTIVIF | |
|  | CA46 | 11653 | 41.80 | IGKV3-20(493) | | IGKJ1(141) | CQQYGSSPPWTF | |
|  |  | 6183 | 22.18 | IGKV2-28(435.2) | | IGKJ2(161.5) | CMQALQTPHTF | |
|  |  | 2969 | 10.65 | IGHV5-51(391.4) | IGHD5-12(26), IGHD2-21(25), IGHD3-22(25) | IGHJ4(170.1) |  | |
|  |  | 2944 | 10.56 | IGHV5-51(391.9) | IGHD3-22(30), IGHD5-12(26) | IGHJ4(170.9) | CARFDRGGDYW | |
|  |  | 2731 | 9.80 | IGHV5-51(363) | IGHD3-22(30), IGHD3-9(26), IGHD5-12(26) | IGHJ4(163.9) | CARARFDRGGDYW | |
|  |  | 449 | 1.61 | IGKV3-20(481.4) | | IGKJ1(138.7) | CQQYRSSPPWTF | |
|  |  | 446 | 1.60 | IGKV3-20(617) | | IGKJ1(114.5) | CQQYGSSPPWTF | |
|  | P3HR-1 | 2373 | 74.39 | IGKV3-15(299) | | IGKJ4(113.5) | CQQFDYWPPPTF | |
|  |  | 215 | 6.74 | IGHV3-30(210.3) | IGHD2-15(33), IGHD2-2(33), IGHD3-9(33) | IGHJ4(187) | CAISGSGIFSLPLFDYW | |
|  |  | 31 | 0.97 | IGKV3-31(50) | | IGKJ5(155.3) | CA_TF |  |
| CLL-small lymphocytic lymphoma | JVM-3 | 104390 | 67.34 | IGKV1-5(448) | | IGKJ1(161.4) | CQQYNSYPWTF | |
|  |  | 40194 | 25.93 | IGHV3-7(304.5) | IGHD3-22(50) | IGHJ4(200.1) | CARVSIQDDSSRYFDYW | |
|  |  | 9877 | 6.37 | IGKV2-29(447) | | IGKJ4(164.8) | *MQGIH_SGLTF | |
|  | MEC-1 | 22494 | 71.17 | IGKV4-1(462.6) | | IGKJ2(155.5) | CQQYYSIPYTF | |
|  |  | 8543 | 27.03 | IGHV4-59(231.1), IGHV4-61(212.7), IGHV4-4(197.4) | IGHD3-9(30) | IGHJ4(169.4) | CARSQGVLTAIDYW | |
|  | JVM-2 | 27509 | 63.68 | IGLV2-14(396) | | IGLJ2(156.9) | CSSYTSSSVVF | |
|  |  | 15234 | 35.27 | IGHV3-9(336.9) | IGHD6-19(46) | IGHJ4(177.9) | CAKDMQGAVAGGVDYW | |
|  | EHEB | 83657 | 79.94 | IGKV1-33(369.8) | | IGKJ2(123.5) | CQQYDGLPRTF | |
|  |  | 20482 | 19.57 | IGHV1-18(213.5) | IGHD4-17(50), IGHD4-23(45) | IGHJ5(148), IGHJ4(145.5) | CARDDGGGKGDYGRLW | |
|  |  | 42 | 0.04 | IGKV1-33(404.2) | | IGKJ2(140.9) | GLPRTF |  |
| DLBCL | DOHH-2 | 12436 | 67.43 | IGLV2-11(314.9) | | IGLJ3(136.3), IGLJ2(131.3) | CCSYAGGYSLGVF | |
|  |  | 4852 | 26.31 | IGHV3-15(261) | IGHD1-26(40), IGHD2-15(35) | IGHJ4(152.8), IGHJ5(142.1) | CTTAPTLELPCDFW | |
|  |  | 275 | 1.49 | IGKV1-8(447.6) | | IGKJ3(154.5) | CQQYYS_PPFTF | |
|  |  | 256 | 1.39 | IGLV3-19(297.9) | | IGLJ3(145.3) | YNSRVSSGCTWVF | |
|  |  | 184 | 1.00 | TRAV19(49.4) | | TRAJ24(32.4) | CALS_GG*G | |
|  | Pfeiffer | 74652 | 89.56 | IGKV1-5(368.7) | | IGKJ2(155.9) | CQQNNSYPYTF | |
|  |  | 3396 | 4.07 | IGHV3-7(366.3) | IGHD4-17(26), IGHD4-23(26), IGHD1-1(25) | IGHJ6(155.6) | CGRRYNGMDVW | |
|  |  | 2291 | 2.75 | IGKV1-5(275) | | IGKJ2(136.3) | CHHYKTHSF | |
|  |  | 2117 | 2.54 | IGKV1-5(287.4) | | IGKJ2(135.3) | CHQYKTHSF | |
|  |  | 196 | 0.24 | IGLV8-61(60.4) | | IGLJ7(138.8) | CV_VF |  |
|  |  | 62 | 0.07 | IGKV3-31(49.9) | | IGKJ5(168) | CG_TF |  |
|  | U-937 | 36 | 6.23 | TRAV8-7(68.2) | | TRAJ19(55.1) | CAGADRLQTGMRGAF | |
|  | A4/Fuk | 35456 | 70.78 | IGKV1-27(394.7) | | IGKJ4(132) | CQKYNSAPLTF | |
|  |  | 14129 | 28.21 | IGHV4-34(230.4) | IGHD3-10(69) | IGHJ6(104.4) | CASLRFGDYYHGLGVW | |
|  | SU-DHL-6 | 19719 | 80.55 | IGKV1-5(394.1) | | IGKJ1(122.7) | CQQYQSYSWTF | |
|  |  | 4046 | 16.53 | IGHV3-48(189.2) | IGHD3-16(31), IGHD2-15(30), IGHD2-21(30) | IGHJ4(123.5) | CATGGTDSTRGLLYW | |
|  |  | 57 | 0.23 | IGKV3-20(313.4) | | IGKJ4(128.3) | CQQYAGSALTF | |
|  |  | 46 | 0.19 | IGKV3-31(51.1) | | IGKJ5(168.8) | CG_TF |  |
|  | WSU-DLCL2 | 5825 | 70.11 | IGLV2-14(157.2), IGLV2-23(131.8) | | IGLJ2(97.6), IGLJ3(97.6) | CCSYTEDSTYIF | |
|  |  | 1442 | 17.36 | IGHV2-26(166.3), IGHV2-5(149), IGHV2-70(140.2) | IGHD3-9(46), IGHD2-8(43), IGHD2-21(40) | IGHJ5(159.9) | CARVKVTISGSNWFDSW | |
|  |  | 624 | 7.51 | IGKV2-30(243.6) | | IGKJ5(85.7) | CMEGT_LAPL | |
|  |  | 59 | 0.71 | IGKV3-20(314.2) | | IGKJ4(123.8) | CQQYAGSALTF | |
|  |  | 36 | 0.43 | TRAV8-2(53.5) | | TRAJ26(69.7) | CVVGITMIDLYPRDSWNFVF | |
|  | DB | 3521 | 52.19 | IGLV2-8(139.6) | | IGLJ2(65.9), IGLJ3(65.9) | CSSFGRSNIVLF | |
|  |  | 2396 | 35.52 | IGHV4-39(79.1), IGHV7-81(65.4) | IGHD6-25(31), IGHD4-17(30), IGHD4-23(30) | IGHJ3(57.5) | CAGRLRGLFSFEYW | |
|  |  | 61 | 0.90 | TRAV8-7(67.8) | | TRAJ19(54.1) | CAGADRLQTGMRGAF | |
|  | Toledo | 11815 | 58.54 | IGLV2-14(392.6) | | IGLJ7(143.8) | CSSYTS_QHSVF | |
|  |  | 6830 | 33.84 | IGLV3-21(409.2) | | IGLJ6(159) | CQVWDSS_*SPNVF | |
|  |  | 316 | 1.57 | IGLV8-61(59.9) | | IGLJ7(137.5) | CV_VF |  |
|  |  | 114 | 0.56 | IGLV2-23(433.2) | | IGLJ7(122.3) | CCSYAGSSTFVF | |
|  |  | 82 | 0.41 | TRAV8-7(67.7) | | TRAJ19(55.1) | CAGADRLQTGMRGAF | |
|  |  | 42 | 0.21 | IGKV3-20(228.9) | | IGKJ4(118.8) | CQQYAGSALTF | |
|  | KARPAS-422 | 17537 | 96.07 | IGKV2-28(427.8) | | IGKJ4(143.9) | CMQALQTVTF | |
|  |  | 162 | 0.89 | IGHV4-61(135.8), IGHV4-4(131.3), IGHV4-59(128.7) | IGHD3-10(65) | IGHJ6(47.3) | CAKNSSVPIVRGLNKRYSFFDLW | |
|  |  | 43 | 0.24 | IGKV1-33(87.3) | | IGKJ3(87) | CHWHE_ISVTL | |
|  | NU-DHL-1 | 17821 | 41.47 | IGLV3-1(403.7) | | IGLJ1(153.6) | CQSWDNSTYVF | |
|  |  | 9521 | 22.15 | IGHV3-53(120.3) | IGHD1-14(35), IGHD2-21(30) | IGHJ3(115.7) | CARENGTGRGPQALDLW | |
|  |  | 8568 | 19.94 | IGLV2-5(307.1) | | IGLJ3(149.5) | CCSYTAGVTWVF | |
|  |  | 6523 | 15.18 | IGKV1-39(300.9) | | IGKJ5(122.9) | CQQNYRTPIAC | |
|  | OCI-LY3 | 10491 | 88.26 | IGLV3-21(283.7) | | IGLJ3(129.5) | CQVWDNSGGQLWMF | |
|  |  | 275 | 2.31 | IGHV4-34(130.4) | IGHD6-19(45), IGHD3-10(41), IGHD3-22(41) | IGHJ1(79.9) | CARGRTGDAEGDVAGLGYYYDFW | |
|  |  | 51 | 0.43 | TRAV8-7(67) | | TRAJ19(55) | CAGADRLQTGMRGAF | |
|  |  | 33 | 0.28 | TRAV14DV4(76) | TRDD2(25) | TRAJ4(44) | CAVR_GGRG | |
|  | SU-DHL-5 | 1779 | 50.63 | IGHV1-8(171.4), IGHV1-2(164.7) | IGHD2-21(36), IGHD2-15(35), IGHD3-3(35) | IGHJ4(142.2) | CARGPNLMSGECPYNFAYW | |
|  |  | 1386 | 39.44 | IGLV1-44(265.9), IGLV1-36(225.5) | | IGLJ2(96.2), IGLJ3(94.6) | CATWDDTLNGVVF | |
|  |  | 56 | 1.59 | IGKV3-20(317.4) | | IGKJ4(121.8) | CQQYAGSALTF | |
|  | OCI-LY-19 | 6917 | 80.76 | IGLV1-40(476.6) | | IGLJ3(154.5) | CQSYDSSLSGSWVF | |
|  |  | 1103 | 12.88 | IGHV3-48(124), IGHV3-21(100.4) | IGHD1-1(25), IGHD2-15(25), IGHD2-21(25) | IGHJ4(104.3) | CARSGAQHYFEKW | |
|  |  | 41 | 0.48 | IGLV11-55(79.3) | | IGLJ7(40) | CAMG_PQF | |
|  |  | 35 | 0.41 | TRAV8-7(67) | | TRAJ19(55) | CAGADRLQTGMRGAF | |
|  | SU-DHL-4 | 15597 | 75.84 | IGKV2-28(411.3) | | IGKJ4(152.1) | CMQAVQTPLTF | |
|  |  | 4375 | 21.27 | IGHV4-4(189.2) | IGHD3-16(40), IGHD5-12(35) | IGHJ4(168.5) | CARRSPDYGHNFDFW | |
|  |  | 57 | 0.28 | TRAV8-7(68) | | TRAJ19(55) | CAGADRLQTGMRGAF | |
|  |  | 42 | 0.20 | TRAV8-2(57.6) | | TRAJ26(65.1) | CVVGITMIDLYPRDSWNFVF | |
|  |  | 39 | 0.19 | IGKV3-20(295.1) | | IGKJ4(112.3) | CQQYAGSALTF | |
|  | SU-DHL-8 | 50490 | 68.64 | IGLV1-44(453.3), IGLV1-47(367.2) | | IGLJ3(151.2) | CAAWDDSLNGWVF | |
|  |  | 18013 | 24.49 | IGKV1-39(182.9), IGKV1-8(148.4) | | IGKJ1(39.7), IGKJ2(32.5) | CQQSFSFPLTF | |
|  |  | 4340 | 5.90 | IGHV3-53(127.2) | IGHD4-17(36), IGHD2-21(31) | IGHJ4(159.1) | CARRAVTTFADYFDSW | |
|  |  | 51 | 0.07 | TRAV8-7(67) | | TRAJ19(55) | CAGADRLQTGMRGAF | |
|  |  | 34 | 0.05 | IGKV3-20(313.4) | | IGKJ4(116.5) | CQQYAGSALTF | |
|  | SU-DHL-10 | 4565 | 49.82 | IGHV7-81(75) | IGHD6-13(30), IGHD6-19(30), IGHD6-6(30) | IGHJ2(150) | CARGRTRTFFDLW | |
|  |  | 3834 | 41.84 | IGLV2-23(244.7) | | IGLJ2(100.3) | CCSYTGRSPHVVF | |
|  |  | 182 | 1.99 | TRAV8-7(67.3) | | TRAJ19(55) | CAGADRLQTGMRGAF | |
|  |  | 61 | 0.67 | IGKV3-20(342.5) | | IGKJ4(128.1) | CQQYAGSALTF | |
| Hodgkin lymphoma | L-540 | 56 | 9.91 | TRAV8-4(297.4) | | TRAJ9(232) | CAVKGGGFKTIF | |
|  | TO 175.T | 47 | 9.46 | IGKV3-20(274.2) | | IGKJ4(115.3) | CQQYAGSALTF | |
|  | HD-MY-Z | 36 | 5.12 | TRAV8-7(67) | | TRAJ19(55) | CAGADRLQTGMRGAF | |
|  |  | 32 | 4.55 | IGLV4-60(75) | | IGLJ1(45) | CETWGVNG_DGYFGGHE | |
|  | KM-H2 | 878 | 61.23 | IGKV4-1(288.6) | | IGKJ4(149.3) | CHQYYSAPLTF | |
|  |  | 41 | 2.86 | TRAV8-7(69.2) | | TRAJ19(55) | CAGADRLQTGMRGAF | |
|  | L-1236 | 34 | 5.61 | TRAV8-7(67) | | TRAJ19(55) | CAGADRLQTGMRGAF | |
|  | Hs 611.T | 2517 | 26.69 | IGKV1-39(449.9) | | IGKJ1(163.1) | CQQSYSTSWTF | |
|  |  | 2203 | 23.36 | IGHV4-61(234.5), IGHV4-4(233.9), IGHV4-34(194.2), IGHV4-59(193.7) | IGHD6-6(59) | IGHJ4(180.4) | CARGEYSNSPLFDYW | |
|  |  | 796 | 8.44 | IGKV1-39(442.5) | | IGKJ1(162.7) | CQQSYNTSWTF | |
|  |  | 339 | 3.59 | IGHV4-61(227.3), IGHV4-4(223.4), IGHV4-34(203.5), IGHV4-59(189.5) | IGHD6-6(73) | IGHJ4(177.5) | CARGEYSSSPLFDYW | |
|  |  | 287 | 3.04 | IGHV4-4(234.3), IGHV4-61(233.2) | IGHD6-6(50) | IGHJ4(182.7) | CARGEYSNSPLFDYW | |
|  |  | 250 | 2.65 | IGHV4-4(232.9), IGHV4-61(228), IGHV4-59(192.8) | IGHD6-6(45) | IGHJ4(182.8) | CARGEYSNSPLFDYW | |
|  |  | 204 | 2.16 | IGHV4-4(215), IGHV4-61(196.9), IGHV4-34(180.8) | IGHD6-6(59) | IGHJ4(179.8) | CVRGEYSNSPLFDYW | |
|  |  | 170 | 1.80 | IGKV1-39(417.5) | | IGKJ1(164) | CQQTYSTSWTF | |
|  |  | 162 | 1.72 | IGKV1-39(442.1) | | IGKJ1(157.5) | CQQSYTTSWTF | |
|  |  | 138 | 1.46 | IGKV1-39(444.4) | | IGKJ1(162.9) | CQQNYSTSWTF | |
|  |  | 131 | 1.39 | IGKV1-39(461.2) | | IGKJ1(160.1) | CQQSYSSSWTF | |
|  |  | 126 | 1.34 | IGHV4-4(226.4), IGHV4-61(213.3), IGHV4-34(206), IGHV4-59(182.9) | IGHD6-6(59) | IGHJ4(191.4) | CARGEYSNSPLFDYW | |
|  |  | 121 | 1.28 | IGKV1-39(428.2) | | IGKJ1(158.7) | CQQSSSTSWTF | |
|  |  | 71 | 0.75 | IGHV4-61(205.4), IGHV4-4(205.2) | IGHD4-11(51), IGHD4-4(51) | IGHJ4(184.4) | CARGDYSNSPLFDYW | |
|  |  | 56 | 0.59 | IGKV1-39(478) | | IGKJ1(154.9) | CQQSYSISWTF | |
|  |  | 31 | 0.33 | IGKV1-39(421.7) | | IGKJ1(170.2) | CQQTYNTSWTF | |
|  | L-428 | 33 | 6.99 | IGKV2-40(421.9) | | IGKJ4(147.5) | SMQLF |  |
| Mantle cell lymphoma | JeKo-1 | 40081 | 82.20 | IGKV3-20(504.8) | | IGKJ2(149.7) | CQQYGSSPNTF | |
|  |  | 7342 | 15.06 | IGHV2-70(302.6) | IGHD3-3(70) | IGHJ4(164) | CARIRGFGVVNLPDYW | |
|  |  | 832 | 1.71 | IGLV8-61(60.5) | | IGLJ7(138.6) | CV_VF |  |
|  | REC-1 | 107815 | 81.94 | IGKV3-11(494.3) | | IGKJ1(148.3) | CQQPGTF |  |
|  |  | 16871 | 12.82 | IGHV1-2(300) | IGHD2-21(91) | IGHJ5(143), IGHJ4(142) | CARRGEGYCGGDCYSLW | |
|  |  | 6320 | 4.80 | IGKV1-39(460.9) | | IGKJ2(129.8) | CQQSYS_PLVADG | |
|  |  | 36 | 0.03 | TRAV8-7(67) | | TRAJ19(55) | CAGADRLQTGMRGAF | |
|  | GRANTA-519 | 32524 | 51.57 | IGLV4-69(458.9) | | IGLJ2(151) | CQTWGTSVVF | |
|  |  | 19945 | 31.62 | IGHV4-59(295.9), IGHV4-61(292), IGHV4-4(259.7) | IGHD5-24(40), IGHD5-12(35) | IGHJ4(218) | CARVGYRSEYYFDYW | |
|  |  | 10204 | 16.18 | IGLV4-60(406.7) | | IGLJ2(123.4), IGLJ3(123.4) | CETWDS_TPWVF | |
|  | Mino | 30091 | 89.73 | IGLV3-19(403.4) | | IGLJ2(167) | CNSRDSSGNPHVVF | |
|  |  | 2059 | 6.14 | IGHV3-21(209), IGHV3-7(186.3) | IGHD3-10(75) | IGHJ6(123.7) | CVREGGADLLWFGEWDPRGGTHYYGMDVW | |
|  |  | 686 | 2.05 | IGLV8-61(60.1) | | IGLJ7(139.6) | CV_VF |  |
|  |  | 37 | 0.11 | TRAV8-7(69.7) | | TRAJ19(55) | CAGADRLQTGMRGAF | |
| Mycosis fungoides-Sezary syndrome | HuT 78 | 2023 | 55.39 | TRBV13(405.5) | TRBD1(40) | TRBJ1-2(159) | CASSTSPGGRGYTF | |
|  |  | 774 | 21.19 | TRAV8-6(380.7) | | TRAJ37(224.5) | CAVSKGSNTGKLIF | |
|  |  | 154 | 4.22 | TRGV5(391.4) | | TRGJ1(209.2), TRGJ2(209.2) | CATWDRLTYYYKKLF | |
|  |  | 92 | 2.52 | TRAV20(385.2) | | TRAJ24(260.5) | CAVQARDN_DSWGKLQF | |
|  |  | 46 | 1.26 | TRGV8(391.1) | | TRGJP1(216.7) | CATWTPS_GWFKIF | |
|  |  | 32 | 0.88 | TRBV13(650.1) | | TRBJ2-6(40) | CASS_PAQ | |
|  | MJ | 220 | 27.71 | TRBV28(359.7) | TRBD1(30), TRBD2(30) | TRBJ1-1(172.1) | CASSRDDTHEAFF | |
|  |  | 39 | 4.91 | TRAV38-2DV8(323.8) | TRDD3(25) | TRAJ26(232.2) | CAYRSGGYYGQNFVF | |
|  | HuT 102 | 4967 | 53.71 | TRAV12-2(368.4) | | TRAJ22(251) | CAVFFSGSARQLTF | |
|  |  | 2752 | 29.76 | TRBV30(400.6) | TRBD1(31) | TRBJ1-2(158.4) | CAWSRQEAVGGYTF | |
|  |  | 974 | 10.53 | TRAV12-2(397.1) | | TRAJ3(250.6) | CAVNDSSASKIIF | |
| Malignant histiocytosis | DEL | 409 | 50.49 | TRAV40(82.8) | | TRAJ4(48.8) | CLLGSISLGILSQ | |
|  |  | 33 | 4.07 | TRAV8-7(67) | | TRAJ19(55) | CAGADRLQTGMRGAF | |
| Peripheral T cell lymphoma unspecified | SUP-T11 | 6490 | 73.19 | TRBV7-9(321.4) | TRBD1(25) | TRBJ1-1(207.2) | CASSFTGNTEAFF | |
|  |  | 1408 | 15.88 | TRBV21-1(366.7) | TRBD1(45) | TRBJ1-5(225) | CASSKDRGHSNQPQHF | |
|  |  | 282 | 3.18 | TRAV25(370.7) | | TRAJ40(241.1) | CVTSGTYKYIF | |
|  |  | 61 | 0.69 | IGKV3-20(356.8) | | IGKJ4(117.6) | CQQYAGSALTF | |
| Myeloma | KMS-12-BM | 18169 | 96.80 | IGHV3-7(268.6) | IGHD4-23(40), IGHD4-17(35) | IGHJ6(124.5) | CARAGYGGAGAMDVW | |
|  |  | 50 | 0.27 | IGHV3-7(358.4) | IGHD5-18(30), IGHD5-5(30), IGHD2-2(28) | IGHJ2(17), IGHJ2P(14.1) | CARA_IRW | |
|  | NCI-H929 | 117801 | 99.50 | IGKV3-15(428.1) | | IGKJ1(149.2) | CQQYNNWPPWTF | |
|  |  | 53 | 0.04 | TRAV8-7(67) | | TRAJ19(55) | CAGADRLQTGMRGAF | |
|  |  | 37 | 0.03 | IGKV3-31(50) | | IGKJ5(170.8) | CG_TF |  |
|  | KARPAS-620 | 403293 | 99.82 | IGKV3-20(432) | | IGKJ4(132.8) | CQQYAGSALTF | |
|  |  | 95 | 0.02 | IGLV3-24(37.8) | | IGLJ2(150.2), IGLJ3(134.2) | NITW_IALF | |
|  |  | 56 | 0.01 | TRAV8-7(67.7) | | TRAJ19(55.1) | CAGADRLQTGMRGAF | |
|  | KMM-1 | 7983 | 91.92 | IGLV1-51(238.2) | | IGLJ2(97.1), IGLJ3(96.4) | CAAWDSSLTSVIF | |
|  |  | 160 | 1.84 | IGHV1-18(60.4) | IGHD4-17(30), IGHD4-23(26) | IGHJ2(101.3) | CVKSDFSEIHRYFEVW | |
|  |  | 32 | 0.37 | IGKV2-40(346) | | IGKJ4(155.7) | SMQLF |  |
|  | HuNS1 | 27376 | 89.72 | IGKV1-33(397.1) | | IGKJ4(141.8) | CQQHDHLPLTF | |
|  |  | 2581 | 8.46 | IGHV4-31(281.5) | IGHD5-24(75) | IGHJ4(166.7) | CAIGVDGYNYPFFDNW | |
|  | KHM-1B | 505858 | 75.59 | IGLV1-44(400.4), IGLV1-47(347.7) | | IGLJ3(128.1) | CAAWDDSLNEGVF | |
|  |  | 162817 | 24.33 | IGHV1-67(131.9), IGHV1-3(131.1) | IGHD5-24(50) | IGHJ4(136.6), IGHJ5(133.1) | CARGGDGYMLYDSW | |
|  |  | 56 | 0.01 | IGLV3-10(327.6) | | IGLJ3(102.3), IGLJ2(97.1) | CFSTDGTANRGVF | |
|  |  | 36 | 0.01 | IGHV3-23(145.1) | IGHD7-27(35), IGHD1-26(30), IGHD3-3(30) | IGHJ2(158.9) | CARLGRNEWGLEYFDLW | |
|  | MOLP-8 | 119627 | 58.73 | IGLV2-23(237.3) | | IGLJ1(103.3) | CSSFADDYSYVF | |
|  |  | 82270 | 40.39 | IGHV2-5(197.1) | IGHD6-19(31), IGHD6-25(30), IGHD5-24(27) | IGHJ4(111.4) | CAHKGLLEAAGTFDFW | |
|  |  | 974 | 0.48 | IGLV3-4(52.3) | | IGLJ1(111.9) | PRLQM_YSYVF | |
|  |  | 77 | 0.04 | IGHV2-5(220.1) | IGHD3-3(26), IGHD5-24(25) | IGHJ4(39), IGHJ5(39), IGHJ2(38.6), IGHJ2P(35.9), IGHJ3(35.9) | CAHKG_FRGGW | |
|  |  | 75 | 0.04 | TRAV12-1(46) | | TRAJ26(53.9) | CVVSHEDSRTLLNAS_GA*KSAM*QTMAP*N | |
|  |  | 31 | 0.02 | TRAV8-7(68.6) | | TRAJ19(55) | CAGADRLQTGMRGAF | |
|  | L-363 | 355114 | 99.81 | IGLV2-23(373.9) | | IGLJ3(114) | CCSFADPSTLVF | |
|  | AMO-1 | 392010 | 84.00 | IGKV3-20(359.8) | | IGKJ2(148.6) | CQQYGSSPYTF | |
|  |  | 72117 | 15.45 | IGHV3-23(269.7) | IGHD3-16(30), IGHD7-27(27), IGHD3-3(26) | IGHJ4(103.1) | CATGRRKDLGAPLDHW | |
|  |  | 1268 | 0.27 | IGHV3-60(72.7) | IGHD3-16(30), IGHD7-27(27), IGHD3-3(26) | IGHJ4(100.6) | CVRQEGAK_LGAPLDHW | |
|  |  | 443 | 0.09 | IGHV3-23(318) | | IGHJ2(43.8) | CATGR_QRSW | |
|  |  | 99 | 0.02 | IGHV3-23(96.1) | IGHD4-17(55), IGHD4-23(55) | IGHJ6(172.2) | CATGRRKDLGAPLDHWGQGTVVIVSSDVW | |
|  |  | 40 | 0.01 | IGKV3-31(48.8) | | IGKJ5(144.7) | CG_TF |  |
|  |  | 39 | 0.01 | IGHVII-15-1(50.6) | IGHD3-16(25) | IGHJ4(118.6) | KILGP_LDHW | |
|  |  | 32 | 0.01 | TRAV8-7(68.5) | | TRAJ19(55) | CAGADRLQTGMRGAF | |
|  |  | 31 | 0.01 | IGKV1-39(294.3) | | IGKJ1(142.8) | CHQGYSTPWTF | |
|  | SK-MM-2 | 612942 | 99.90 | IGKV1-39(307.2) | | IGKJ1(146) | CHQGYSTPWTF | |
|  |  | 33 | 0.01 | TRAV8-7(68.5) | | TRAJ19(54.9) | CAGADRLQTGMRGAF | |
|  | KMS-27 | 625058 | 61.50 | IGKV2-40(412) | | IGKJ4(151.7) | SMQLF |  |
|  |  | 382112 | 37.60 | IGKV1-39(425.9) | | IGKJ1(161.3) | CQQSYSSPQWTF | |
|  |  | 7645 | 0.75 | IGHV3-48(330.4) | IGHD2-15(25), IGHD2-21(25), IGHD3-16(25) | IGHJ3(169.4) | CARDGGGVAAFDIW | |
|  |  | 956 | 0.09 | IGHV4-39(257.4) | IGHD3-10(88) | IGHJ5(158) | CAIPLGST_GSGSSSW | |
|  |  | 75 | 0.01 | IGKV1-39(337.8) | | IGKJ1(146.1) | CNSVDSSPQWTF | |
|  |  | 46 | 0.00 | TRAV8-7(94.4) | | TRAJ18(41), TRAJ21(40) | GAVLAVLLCDTLLGVTRLEGVIHLPLYF | |
|  | U266B1 | 147989 | 87.81 | IGLV2-8(317) | | IGLJ2(128.9), IGLJ3(128.9) | CSSYAGSNSLIF | |
|  |  | 15670 | 9.30 | IGKV3-20(337.7) | | IGKJ1(143.3) | CQQYG_AHGTF | |
|  |  | 4280 | 2.54 | IGHV1-2(205.8) | IGHD3-3(86) | IGHJ6(130.1) | CAKSDPFWSDYYNFDYSYTLDVW | |
|  |  | 163 | 0.10 | IGHV1-2(363.7) | IGHD3-3(86) | IGHJ4(53.8) | CAKSDPFWSDYYNFDYS | |
|  |  | 59 | 0.04 | IGKV3-20(321.2) | | IGKJ4(117.6) | CQQYAGSALTF | |
|  |  | 40 | 0.02 | IGLV2-8(195.3), IGLV2-11(192.4) | | IGLJ2(124.7), IGLJ3(124.7) | CLRMSCSNSLIF | |
|  |  | 36 | 0.02 | TRAV8-7(68.3) | | TRAJ19(55) | CAGADRLQTGMRGAF | |
|  | JJN-3 | 123120 | 99.28 | IGKV3-15(496.6) | | IGKJ4(137.6) | CQQYNNWPRAF | |
|  |  | 273 | 0.22 | IGKV3-15(353.6) | | IGKJ1(41.1), IGKJ5(41.1) | CQQYNNW_SGFRRG | |
|  | LP-1 | 193100 | 86.94 | IGLV3-21(397) | | IGLJ1(146.9) | CQVWDSTSDHYVF | |
|  |  | 27505 | 12.38 | IGHV3-30(242.2) | IGHD2-8(36), IGHD3-22(35), IGHD5-24(33) | IGHJ6(133.1) | CAKTLLQMGTRGHYYGLDVW | |
|  |  | 797 | 0.36 | IGHV3-30(476.7), IGHV3-33(453.2) | IGHD2-8(36), IGHD3-22(35) | IGHJ3(41.3) | CAKT_YYRW | |
|  | RPMI 8226 | 128512 | 99.58 | IGLV2-14(300) | | IGLJ3(113.4) | CSSYRGSATFEVVF | |
|  |  | 58 | 0.04 | TRAV35(66.7) | | TRAJ15(22.6) | CMIVGSPELTF | |
|  | MM1-S | 211289 | 99.59 | IGLV2-14(347.7) | | IGLJ1(145.9) | CSSYTTSSTYIF | |
|  |  | 115 | 0.05 | IGHV3-30(275), IGHV3-33(264.2) | IGHD2-2(105) | IGHJ6(168.1) | CARDLRG*GERFLVCSSTSCYEDSYYYDMDVW | |
|  |  | 68 | 0.03 | IGLV7-35(47.1) | | IGLJ1(171.5) | LITAARI_TSSTYIF | |
|  | KMS-28BM | 63869 | 98.96 | IGLV3-10(276.5) | | IGLJ3(121.6), IGLJ2(121.5) | CYSTDTSGNQRVF | |
|  |  | 51 | 0.08 | IGKV1-5(410.1) | | IGKJ2(124.4) | CQQYKTYTF | |
|  | KMS-11 | 175273 | 89.33 | IGKV3-15(462.2) | | IGKJ5(135.5) | CQQYDDWPLTF | |
|  |  | 20348 | 10.37 | IGKV1-37(406) | | IGKJ4(113.5) | GQRTYN_PFLTF | |
|  |  | 46 | 0.02 | IGLV4-60(75) | | IGLJ1(45) | CETWGVNG_DGYFGGHE | |
|  | KMS-34 | 146773 | 99.56 | IGKV3-15(420.3) | | IGKJ3(136.3) | CQQYKTWLPLTF | |
|  | OPM-2 | 144947 | 99.66 | IGLV3-25(377.2), IGLV3-16(306) | | IGLJ3(130.2) | CQSADLSGTVF | |
|  | KMS-21BM | 126658 | 67.54 | IGLV3-10(293.5) | | IGLJ3(110.3), IGLJ2(106.1) | CFSTDGTANRGVF | |
|  |  | 59992 | 31.99 | IGHV3-23(148.4), IGHV3-53(126.5) | IGHD7-27(35), IGHD1-26(30), IGHD3-3(30) | IGHJ2(181.9) | CARLGRNEWGLEYFDLW | |
|  |  | 135 | 0.07 | IGLV1-44(412.4), IGLV1-47(365.2) | | IGLJ3(130.3) | CAAWDDSLNEGVF | |
|  |  | 92 | 0.05 | IGHV3-41(159) | IGHD7-27(35), IGHD1-26(30), IGHD3-3(30) | IGHJ2(210.3) | FIIVRGLEET_WGLEYFDLW | |
|  |  | 45 | 0.02 | IGHV1-67(144.4), IGHV1-3(141.9) | IGHD5-24(50) | IGHJ5(124.4), IGHJ4(124.1) | CARGGDGYMLYDSW | |
|  | KMS-20 | 130874 | 73.21 | IGKV3-15(419.4) | | IGKJ1(135) | CQQYNNWPPLWTF | |
|  |  | 47347 | 26.49 | IGHV4-39(194) | IGHD1-14(43) | IGHJ4(78.8) | CARQSGSSVITYFDLW | |
|  |  | 36 | 0.02 | IGKV1-5(397.4) | | IGKJ2(115.7) | CQQYKTYTF | |
|  | KMS-26 | 396680 | 98.34 | IGKV1-5(438) | | IGKJ2(124.9) | CQQYKTYTF | |
|  |  | 6052 | 1.50 | IGKV2-30(377) | | IGKJ3(149.7) | CMQSIY_ASLTF | |
|  | MOLP-2 | 422942 | 96.49 | IGLV2-11(251.1) | | IGLJ6(39.5) | CCSISPTFSSFVF | |
|  |  | 14611 | 3.33 | IGHV3-74(80.4) | IGHD3-3(56) | IGHJ6(119.6) | CARGEAYDSWSDHWDVW | |
|  |  | 53 | 0.01 | IGKV4-1(589) | | IGKJ4(149.7) | CQQYYSTPLRLTF | |
|  |  | 44 | 0.01 | TRAV13-1(91.8) | | TRAJ34(27.7) | CARGEAY_ILGAII | |
|  | EJM | 397799 | 74.06 | IGLV1-40(466.7) | | IGLJ2(132.6) | CQSYDGSLSAVVF | |
|  |  | 135908 | 25.30 | IGHV2-5(320.2) | IGHD3-22(38), IGHD2-8(36), IGHD3-10(35) | IGHJ4(184.5) | CAHFPSPTSDNNGYYFDYW | |
|  |  | 2525 | 0.47 | IGLV4-3(436.9) | | IGLJ2(132), IGLJ3(132) | CGESHTIDGQVG*VF | |
|  |  | 278 | 0.05 | IGKV4-1(536.6) | | IGKJ4(156.7) | CQQYYS_LRLTF | |
| Gastric cancer or MM | KE-97 | 19374 | 87.92 | IGLV3-21 (362.3) | | IGLJ1(129.1) | CQVWHSRSDHYVF | |
|  |  | 2129 | 9.66 | IGHV1-46 (219) | IGHD3-10 (40) | IGHJ4 (122.1) | CARDSYGSYRQGESDMGYW | |

The number inside the () indicates the score of the alignment.

Yellow color highlighted Asterisks “*” indicated stop codon. Underscores “_” indicated out of frame CDR3 translation.

**Additional file 1: Table S3. 8 rearrangements highly expressed as dominant rearrangements (> 90% fraction) in their corresponding cell lines.**

| Barcode | Count | Fraction % | All V hits | All J hits | All C hits | Amino acids sequence of CDR3 |
| --- | --- | --- | --- | --- | --- | --- |
| ERR188344 | 7483 | 95 | IGKV3-20(386.7) | IGKJ2(146.7) | IGKC(118.8) | CQQYASSSYTF |
| ERR188426 | 9525 | 95 | IGKV1-27(339.4) | IGKJ3(114.3) | IGKC(145.6) | CQRYNIAPPAF |
| ERR188311 | 12524 | 95 | IGLV3-19(228.5) | IGLJ1(136.8) | IGLC1(206.2) | CCSRDSNVNRYVF |
| ERR188388 | 8282 | 94 | IGLV3-1(327.2) | IGLJ3(123) | IGLC3(164.1),  IGLC2(163.9),  IGLC7(148) | CQAWDNSFWVF |
| ERR188324 | 42274 | 94 | IGKV1-17(388.5) | IGKJ2(146.9) | IGKC(122.5) | CLQHNSYPYTF |
| ERR188274 | 7678 | 93 | IGKV2-28(361.8) | IGKJ4(158.6) | IGKC(145.8) | CMQALQTPVTF |
| ERR188051 | 6328 | 91 | IGKV2-30(305.8) | IGKJ2(111.6) | IGKC(192.8) | CFQGTHWPLLTF |
| ERR188027 | 9281 | 90 | IGKV1-33(263.7) | IGKJ3(97.9) | IGKC(164.7) | CQQYDDLITF |

The number inside the () indicates the score of the alignment.

In total, 9,827 different rearrangements of IGH/IGK/IGL (with a cut off of at least 100 sequencing reads, here after referred as index rearrangement) was detected in 426 EBV transformed B lymphocytes lines; 8 of them were highly expressed as prominent dominant rearrangements which occupying more than 90% rearrangement fraction in their corresponding cell lines

**Additional file 1: Table S4. Clones with a stop codon in their CDR3 region.**

| EBV lymphocyte lines | Counts | Fraction % | All V hits | All D hits | All J hits | All C hits | Amino acids sequence of CDR3. |
| --- | --- | --- | --- | --- | --- | --- | --- |
| ERR188347 | 134 | 0.6 | IGHV1-17(217) |  | IGHJ4(141.4),  IGHJ5(134.4) | IGHM(216.8) | CVRF_D*W |
| ERR188196 | 238 | 0.8 | IGHV1-58(128.1) | IGHD3-9(35),  IGHD2-21(31),  IGHD3-22(31) | IGHJ4(129.2) |  | CAADIPVA*SCQIPHFDFW |
| ERR188022 | 124 | 1 | IGHV3-11(127.7) | IGHD4-17(85) | IGHJ4(101.3) | IGHG3(159.3) | CARVG*LR*LRADFW |
| ERR188183 | 529 | 1.3 | IGHV3-49(144.9) | IGHD2-21(87) | IGHJ2(138.6) | IGHM(113) | CSRALLWC*LLFGWFFDLW |
| ERR188261 | 555 | 1.3 | IGHV4-39(177.6),  IGHV4-34(145) | IGHD3-3(78) | IGHJ4(109.6) | IGHM(51.7) | CARRMILDV*Y_IFAVVTYVDYW |
| ERR188056 | 310 | 0.7 | IGHV5-51(192.8) | IGHD4-17(73) | IGHJ4(127) | IGHM(143.5) | CARLYFDLR*LRMRHDYW |
| ERR188462 | 152 | 0.3 | IGHVIII-2-1(262.3) | IGHD2-2(55),  IGHD6-13(55) | IGHJ3(148.9) | IGHM(131.5) | *VTQQLVQANAFDIW |
| ERR188385 | 226 | 1.1 | IGKV1-13(235.6),  IGKV1-8(191.4) | | IGKJ4(122.8) | IGKC(162.6) | CQQC_*WSI |
| ERR188293 | 642 | 0.8 | IGKV1-16(394.9) |  | IGKJ2(124.6) | IGKC(153.5) | CQQY_*LFG |
| ERR188136 | 242 | 1.2 | IGKV1-17(348.9) |  | IGKJ3(120.8) | IGKC(192.4) | CLQH_*LP |
| ERR188406 | 374 | 1.3 | IGKV1-39(265.5) |  | IGKJ4(144.6) | IGKC(198.5) | CQ*TYS_PPLTF |
| ERR188351 | 313 | 2 | IGKV1-8(369.8) |  | IGKJ2(137.2) | IGKC(154) | WQQY_*LPF |
| ERR188360 | 130 | 0.5 | IGKV2-28(287) |  | IGKJ1(148.2) | IGKC(204.1) | CM*AL_YAGTF |
| ERR188122 | 140 | 1.5 | IGKV2-28(348.2) |  | IGKJ2(149.3) | IGKC(204.2) | CMQAL_NS*TF |
| ERR188303 | 141 | 0.4 | IGKV2-29(265) |  | IGKJ1(154.4) | IGKC(228.7) | *MQGVH_SSWTF |
| ERR188387 | 126 | 1.2 | IGKV2-29(357.7) |  | IGKJ4(148.5) | IGKC(144.4) | *MQGIHLRPTF |
| ERR188084 | 122 | 0.4 | IGKV2-29(370.7) |  | IGKJ1(138) | IGKC(201.6) | *MQGI_TFTF |
| ERR188131 | 352 | 0.9 | IGKV2-30(176.7) |  | IGKJ3(148.6) | IGKC(226.4) | CMRAT_T*FTF |
| ERR188370 | 195 | 1.5 | IGKV3-11(334.9) |  | IGKJ5(147.7) | IGKC(160.5) | CQQRSN*ITF |
| ERR188464 | 119 | 1.2 | IGKV3-7(431) |  | IGKJ1(135.9) | IGKC(120.8) | CQQDYN_*AWTF |
| ERR188450 | 188 | 2.1 | IGKV4-1(355.6) |  | IGKJ1(130.3) | IGKC(136.2) | CQQYY_*SWTF |
| ERR188451 | 228 | 2.2 | IGKV4-1(358.9) |  | IGKJ3(150.8) | IGKC(151.7) | CQQY*S_SLFTF |
| ERR188472 | 108 | 0.3 | IGKV7-3(409.9) |  | IGKJ1(148.6) | IGKC(112.4) | CLQSK_FS*TF |
| ERR188072 | 181 | 1.1 | IGLV1-40(338.6) |  | IGLJ2(121.2),  IGLJ3(116.2) | IGLC3(177.3),  IGLC2(177.2),  IGLC7(149.5) | CQSYDS_A*VVPF |
| ERR188433 | 942 | 8.8 | IGLV1-44(208.8) |  | IGLJ3(122.4) | IGLC3(218.7),  IGLC2(218.5),  IGLC7(181.9) | CVTWDDS_*MSWVF |
| ERR188114 | 226 | 1 | IGLV1-44(368.2) |  | IGLJ3(124.8) | IGLC3(143.6),  IGLC2(143.1) | CAPWDDS_*MLNWVF |
| ERR188391 | 627 | 5.6 | IGLV1-47(182.7) |  | IGLJ3(106.8),IGLJ2(87.4) | IGLC3(157.1),  IGLC2(156.9),  IGLC7(142.4) | CAPGDGS_*VVRLF |
| ERR188297 | 203 | 1.8 | IGLV1-47(235) |  | IGLJ3(118.9) | IGLC3(204.5),  IGLC2(204.1),  IGLC7(174.8) | CAAWDDS_*VGWAF |
| ERR188084 | 198 | 0.6 | IGLV1-47(367.4) |  | IGLJ3(121.3) | IGLC3(197),  IGLC2(194.9),  IGLC7(174.3) | CAAWDDS_*VPWVF |
| ERR188212 | 139 | 0.1 | IGLV1-47(394),  IGLV1-44(367.7) | | IGLJ3(117.8) | IGLC2(164.7),  IGLC3(164.7),  IGLC7(134.5) | CAAWDD_A*FWVF |
| ERR188403 | 111 | 0.4 | IGLV1-47(456.6) |  | IGLJ3(102.6) | IGLC3(130.4),  IGLC2(130.1),  IGLC7(111.8) | CAAWDD_A*VAVF |
| ERR188340 | 309 | 1.5 | IGLV1-51(232.8) |  | IGLJ3(99.8) | IGLC2(214.2),  IGLC3(214.1),  IGLC7(186.1),  IGLC6(172.7) | CGAWNYS_*LLEVVF |
| ERR188318 | 602 | 1.1 | IGLV1-51(312.6) |  | IGLJ2(116.6),IGLJ3(116.6) | IGLC3(247.4),  IGLC2(246.9),  IGLC7(208.6) | CGTWDS_A*VPM |
| ERR188355 | 103 | 0.4 | IGLV1-51(367.9) |  | IGLJ3(108.7) | IGLC3(189.5),  IGLC2(189.3) | CGTWDS_A*GVF |
| ERR188288 | 111 | 0.4 | IGLV2-14(240.2) |  | IGLJ3(101.6),  IGLJ2(99.1) | IGLC3(275.5),  IGLC2(275) | CRSYTS_*HSRVF |
| ERR188404 | 1979 | 9.6 | IGLV2-23(254.7),  IGLV2-11(225),  IGLV2-14(211.7) | | IGLJ3(125.7) | IGLC3(188.4),  IGLC2(188.1),  IGLC7(171.4) | CCSCNC_PF*VF |
| ERR188171 | 219 | 3 | IGLV2-23(341.3) |  | IGLJ3(136.7) | IGLC3(191.1),  IGLC2(190.9),  IGLC7(161.2) | CCSYA_*SWVF |
| ERR188367 | 494 | 6.7 | IGLV2-5(465.2) |  | IGLJ2(106.9) | IGLC3(123.1),  IGLC2(123),  IGLC7(100.9) | CCSYTSGAT*IVVF |
| ERR188231 | 155 | 0.3 | IGLV3-16(340.8) |  | IGLJ1(128.9) | IGLC1(181.5) | CLSADS_WYP*SF |
| ERR188432 | 135 | 0.5 | IGLV3-16(386.4) |  | IGLJ3(116.6) | IGLC3(164.7),  IGLC2(164.2),  IGLC7(156.7) | CLSADSS_VL*GVF |
| ERR188458 | 109 | 0.4 | IGLV3-19(348.8) |  | IGLJ1(144.1) | IGLC1(197.4) | CNSRDS_W*HYVF |
| ERR188400 | 326 | 2.7 | IGLV3-19(440.2) |  | IGLJ3(113) | IGLC3(164.8),  IGLC2(164.6) | CNSRDSS_*PFWVF |
| ERR188056 | 200 | 0.4 | IGLV3-21(281.1) |  | IGLJ2(86.3),IGLJ3(86.3) | IGLC3(251.5),  IGLC2(251.3),  IGLC7(206.6) | CQVWHSS_*SLRVF |
| ERR188306 | 424 | 3.9 | IGLV3-21(306.8) |  | IGLJ3(113.6) | IGLC2(138.9),  IGLC3(138.7),  IGLC7(115.9) | CQVWDST_*LSVVF |
| ERR188219 | 499 | 1.2 | IGLV3-27(267.4) |  | IGLJ2(116),IGLJ3(116) | IGLC3(272.1),  IGLC2(271.5),  IGLC7(223.6) | CNSAAD_TI*VV |
| ERR188327 | 6180 | 25.2 | IGLV3-27(317.3) |  | IGLJ2(118.2) | IGLC2(235.4),  IGLC3(234.8) | CYSAAD_R*GVF |
| ERR188074 | 800 | 1.9 | IGLV4-3(263.2) |  | IGLJ2(120.1) | IGLC2(95.7),  IGLC3(95.6),  IGLC7(80.3) | *GESHTIDG_GSLDHVVF |
| ERR188393 | 3023 | 14.7 | IGLV4-69(343.3) |  | IGLJ1(131.6) | IGLC1(144.3) | CQTWG_LA*VF |
| ERR188176 | 174 | 1.4 | IGLV9-49(356.4) |  | IGLJ3(98.6) | IGLC3(122.2),  IGLC2(122),  IGLC7(105.4) | CGAEHGSGRNFV*GVF |
| ERR188147 | 137 | 0.7 | IGLV9-49(405.2) |  | IGLJ3(98.8),IGLJ2(92.7) | IGLC3(171.8),  IGLC2(171.6),  IGLC7(146) | CGADHGSGSNFV*KF |

* indicates stop codon.

The number inside the () indicates the score of the alignment.

Among 9,827 IGH/IGK/IGL clonotypes (supported by at least 100 reads) identified in 426 EBV transformed B lymphocytes lines, 50 of them have nonproductive stop codon (denoted with “*” in column of “AA seq of CDR3”) in their CDR3 regions.

**Additional file 1: Table S5. TCR clone detected in EBV transformed lymphocyte line ERR188361**

| Sample | Count | Clonotype Fraction % | All V hits | All D hits | All J hits | All C hits | AA. Seq. CDR3 |
| --- | --- | --- | --- | --- | --- | --- | --- |
| ERR188361 | 1955 | 28 | IGLV3-21(279.6) | | IGLJ3(108.7) | IGLC3(250.1),  IGLC2(249.9),  IGLC7(203.3) | CQVWDANSDQWLF |
| ERR188361 | 1406 | 20 | IGHV1-69(94.4) | IGHD1-26(36) | IGHJ3(61.9) | IGHA1(193.7) | CASGSLHTNVGAFLTW |
| ERR188361 | 580 | 8 | IGLV1-44(345.1) | | IGLJ3(131.7) | IGLC3(223.3),  IGLC2(223.1) | CAAWDDSLNAWVF |
| ERR188361 | 464 | 7 | IGKV3-20(374.4) | | IGKJ3(121) | IGKC(160.5) | CQQYGSSPLTF |
| ERR188361 | 357 | 5 | IGHV3-41(115.7) | IGHD1-26(36) | IGHJ3(43.2) | IGHA1(252.9) | CITVPAAPY_TNVGAFLTW |
| ERR188361 | 300 | 4 | IGHV3-30(193.8),  IGHV3-33(155.9) | IGHD6-19(70) | IGHJ4(122.1) | IGHM(159.4) | CAKERVLGAVAGTSYFDYW |
| ERR188361 | 143 | 2 | TRBV7-9(86.1),  TRBV11-1(86),  TRBV7-6(84.9) | | TRBJ2-1(52.9) | IGHA1(0.3),  IGHA2(0.3) | CASGSLHT_RRCFSYL |

The number inside the () indicates the score of the alignment.

Among 9,827 IGH/IGK/IGL clonotypes (supported by at least 100 reads) identified in 426 EBV transformed B lymphocytes lines, ERR188361 had 5 BCR clone and one TCR clone (143 reads).
