# Supplementary material for: Profiling the B/T cell receptor repertoire of lymphocyte derived cell lines
Source: BMC Cancer. 2018 Oct 1;18:940. doi: 10.1186/s12885-018-4840-5 (PMC6167786; doi:10.1186/s12885-018-4840-5)
Supplement: Supplementary file 1 — Figure S1. Expression of CD19/CD20, CD79A/B and RAG1/RAG2 in blood cancer cell lines. X axis: 164 blood cancer cell lines grouped base on the disease types (rectangle color bar below the X axis), each dot in X axis represent one cell line, and the Y axis, expression level (log2 value) of indicated genes. Figure S2. Clonal fraction (filtered by >30 reads) of B-ALL, multiple myeloma, diffuse large B cell lymphoma, Burkitt lymphoma, B cell lymphoma (unspecified) and mantle cell lymphoma base on IGH or IGHK/L. Blue color indicates the clonotype fraction of the most dominant clone, Red color indicate the clonotype fraction of second dominant clone, Yellow color indicate the third dominant clone, while any smaller subclones were aggregated and labelled in grey. Figure S3. Clonal fraction (filtered by >30 reads) of T-ALL and anaplastic large cell lymphoma base on TRCA or TRCB. Blue color indicates the clonotype fraction of dominant clone, red color inidicate the clonotype fraction of second dominant clone, while gray color indicate the third dominant clone. Figure S4. Heatmap showing the usage of IGK/L V genes (A), IGK/L J genes (B), and constant region (C) in 462 samples of EBV transformed normal B lymphocytes. Figure S5. The phylogenetic tree inferred based on the rearrangement of the CDR3 region of IGH, IGK and IGL of EBV transformed B lymphocyte samples ERR188025, ERR188358 and ERR188212. These three cell lines have much higher number of rearrangement types than the other B lymphocyte lines. Clonal Fraction (upper panel) and the read counts (lower panel) of the dominant clone of 462 samples of EBV transformed normal B lymphocytes. (ZIP 1290 kb) [file 12885_2018_4840_MOESM1_ESM.zip › Supplementary Figure 1-5R2.pdf]

Supplementary Fig.1

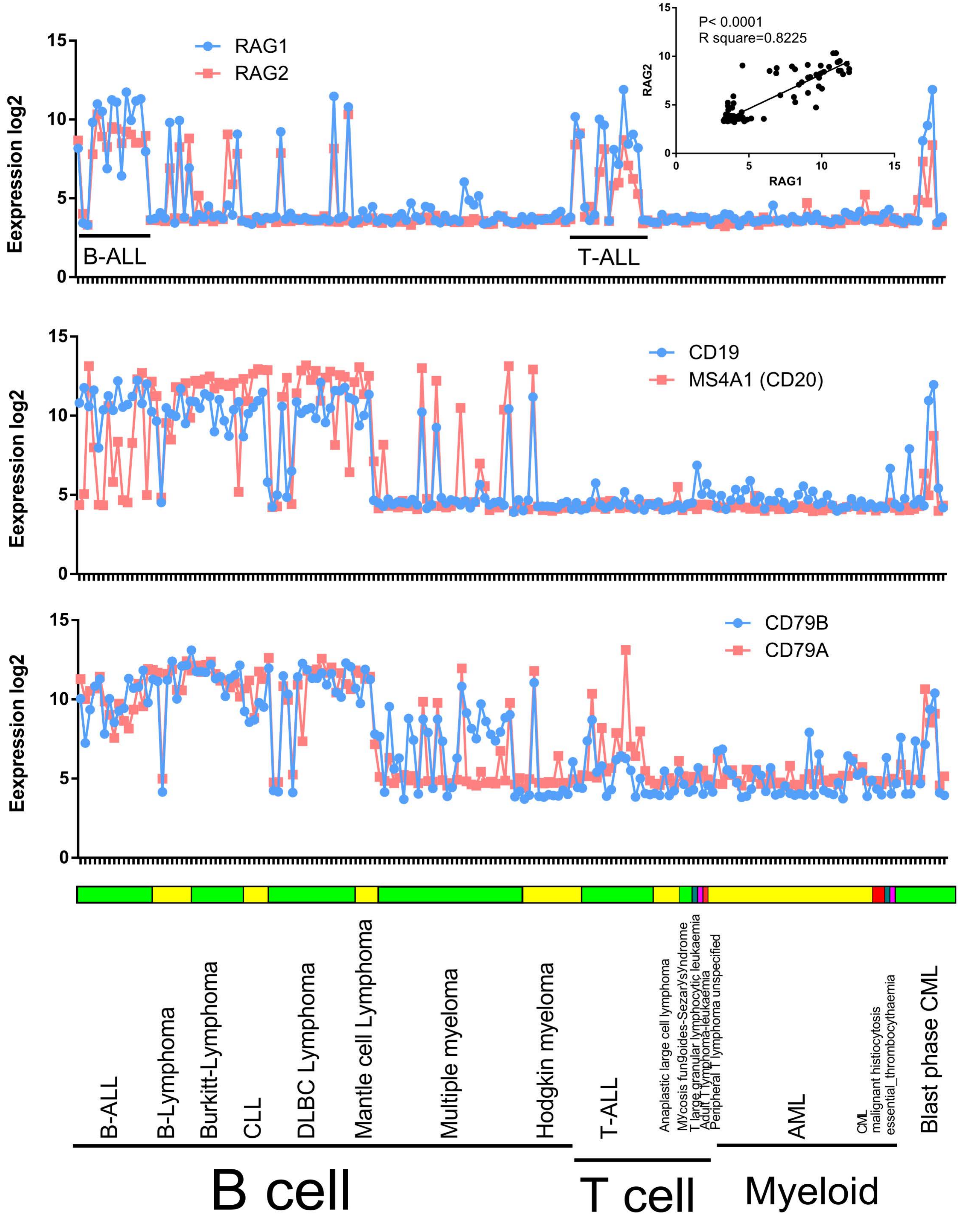

Supplementary Figure 2

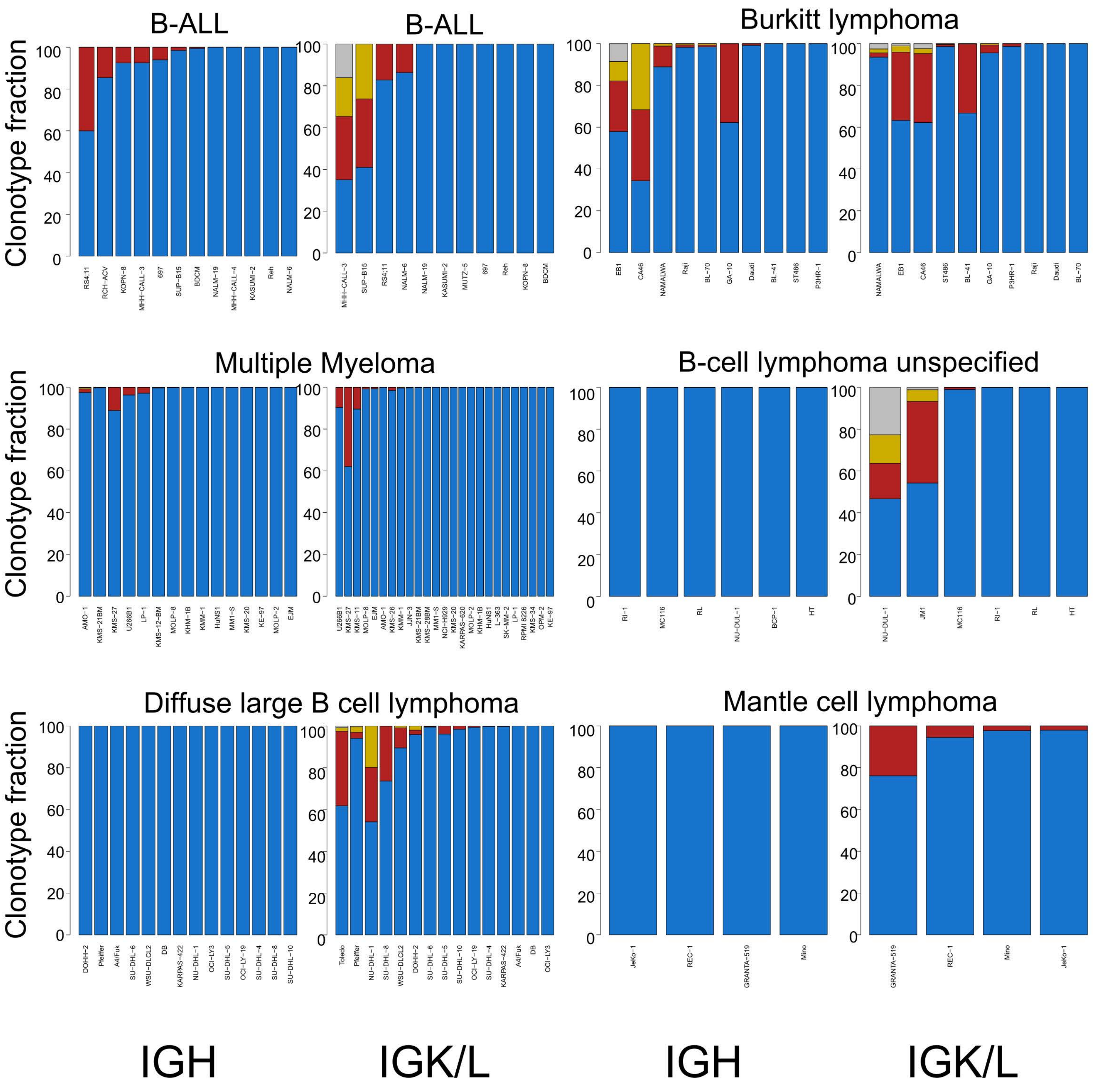

Supplementary Figure 3

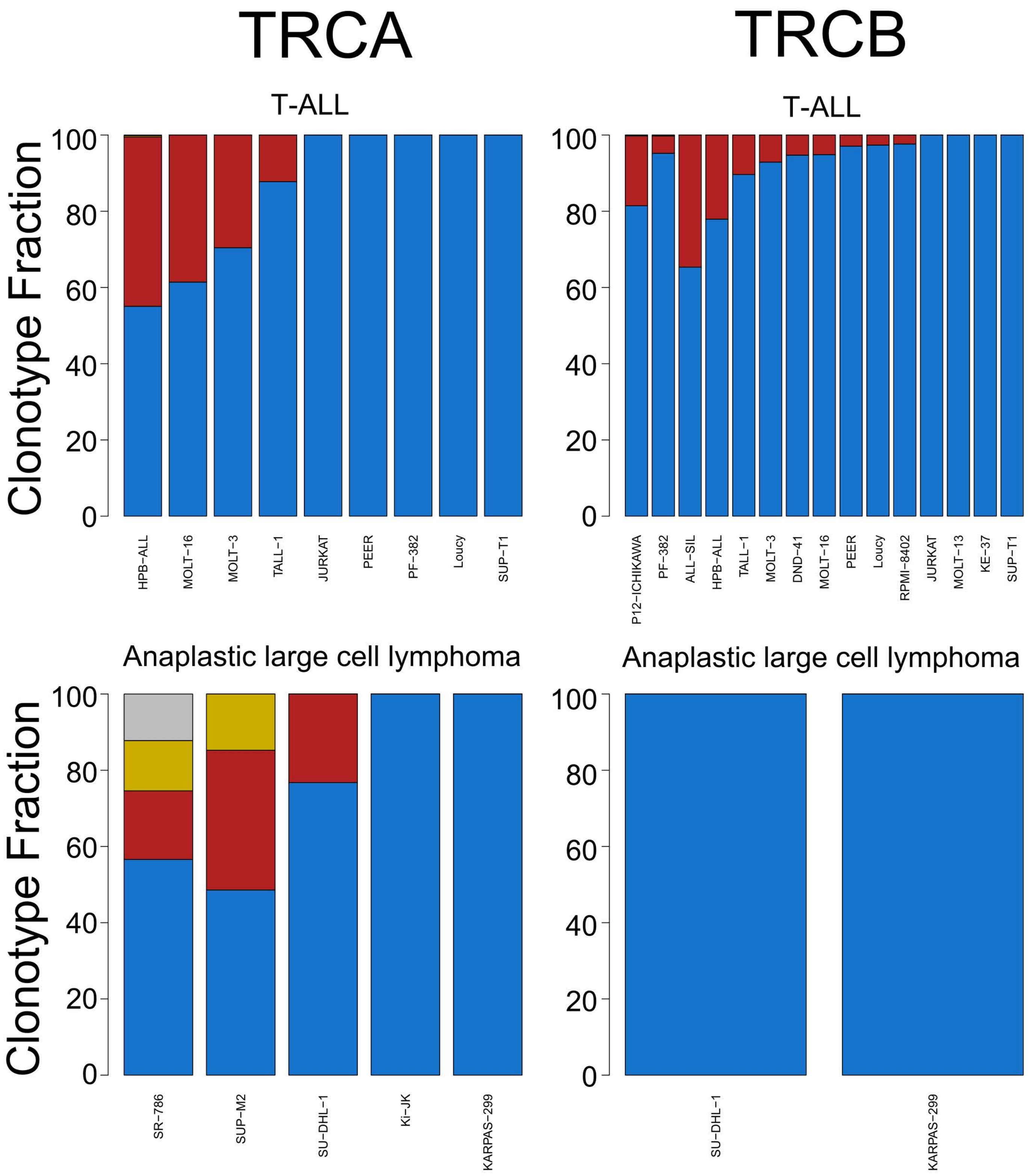

Supplementary Figure 4

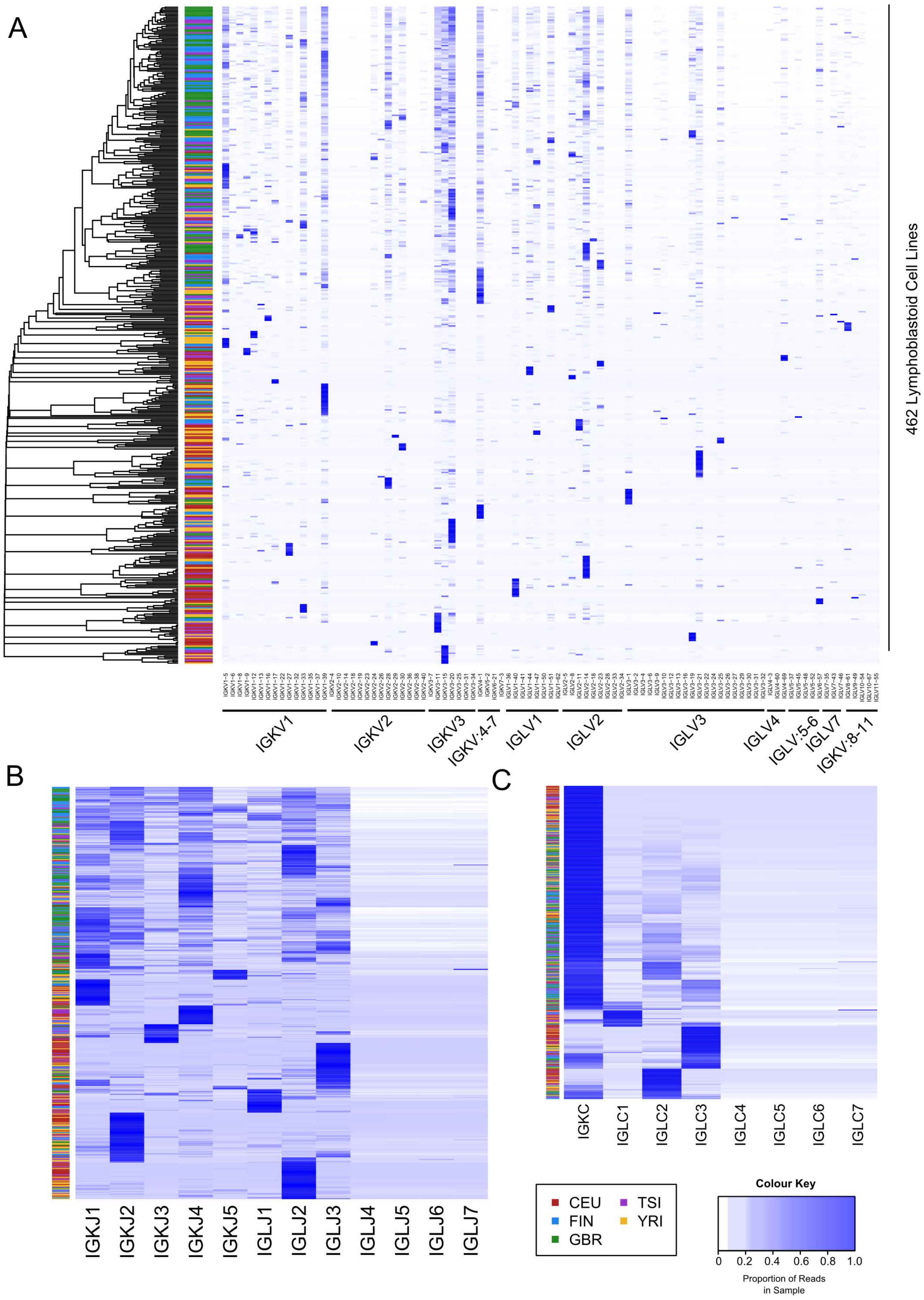

Supplementary Figure 5

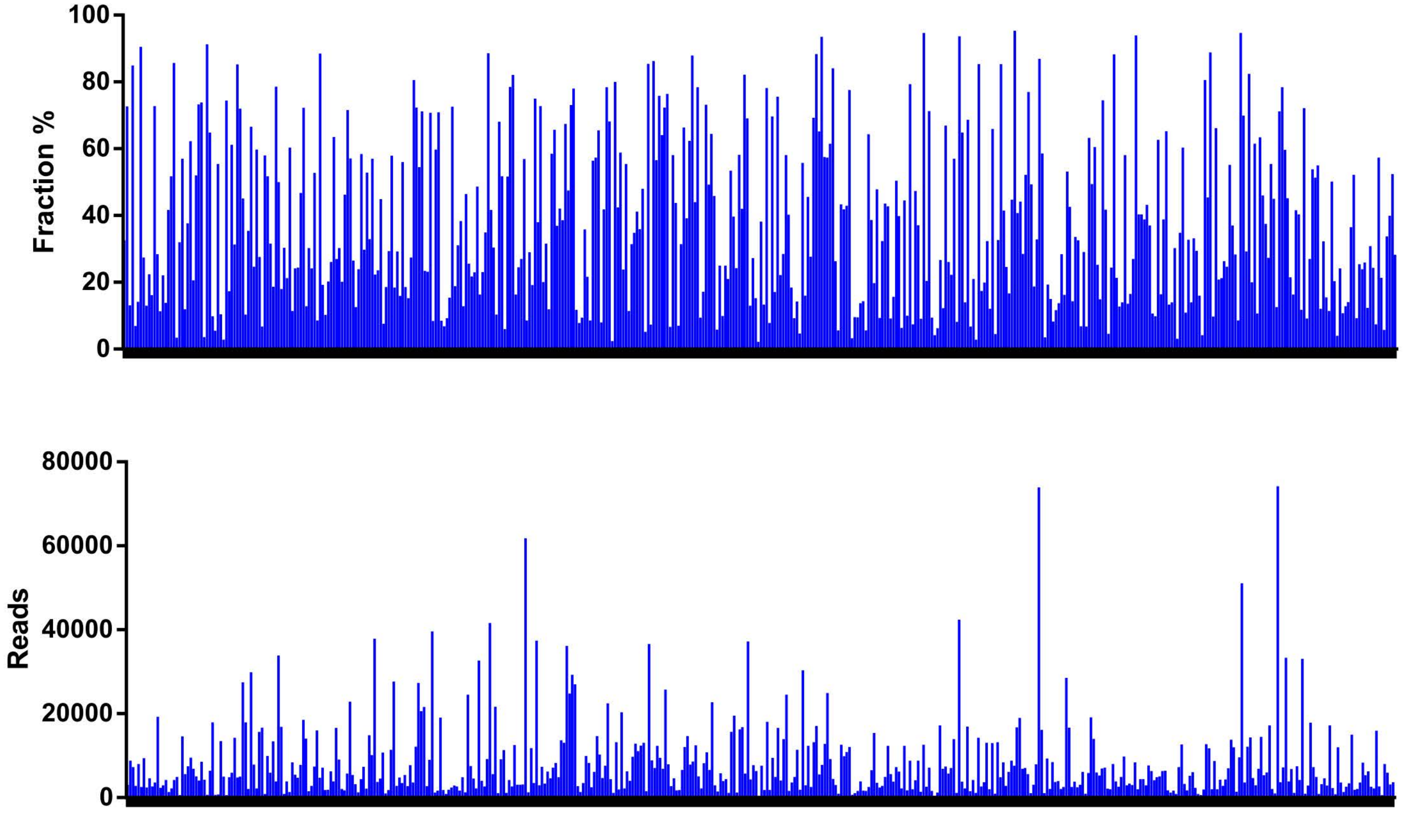

The dominant rearrangement of 462 EBV transformed B lymphocyte cell lines
